# ‘Cold shock’ increases the frequency of homology directed repair gene editing in induced pluripotent stem cells

**DOI:** 10.1038/s41598-018-20358-5

**Published:** 2018-02-01

**Authors:** Q. Guo, G. Mintier, M. Ma-Edmonds, D. Storton, X. Wang, X. Xiao, B Kienzle, D. Zhao, John N. Feder

**Affiliations:** 1 Emerging Technologies and Genomics, Bristol-Myers Squibb Co, Pennington, NJ 08534 USA; 2 Computational Bioinformatics, Bristol-Myers Squibb Co, Pennington, NJ 08534 USA; 3 Lead Discovery and Optimization, Bristol-Myers Squibb Co, Pennington, NJ 08534 USA

## Abstract

Using CRISPR/Cas9 delivered as a RNA modality in conjunction with a lipid specifically formulated for large RNA molecules, we demonstrate that homology directed repair (HDR) rates between 20–40% can be achieved in induced pluripotent stem cells (iPSC). Furthermore, low HDR rates (between 1–20%) can be enhanced two- to ten-fold in both iPSCs and HEK293 cells by ‘cold shocking’ cells at 32 °C for 24–48 hours following transfection. This method can also increases the proportion of loci that have undergone complete sequence conversion across the donor sequence, or ‘perfect HDR’, as opposed to partial sequence conversion where nucleotides more distal to the CRISPR cut site are less efficiently incorporated (‘partial HDR’). We demonstrate that the structure of the single-stranded DNA oligo donor can influence the fidelity of HDR, with oligos symmetric with respect to the CRISPR cleavage site and complementary to the target strand being more efficient at directing ‘perfect HDR’ compared to asymmetric non-target strand complementary oligos. Our protocol represents an efficient method for making CRISPR-mediated, specific DNA sequence changes within the genome that will facilitate the rapid generation of genetic models of human disease in iPSCs as well as other genome engineered cell lines.

## Introduction

One of the most promising applications of the Clustered Regularly Spaced Palindromic Repeats (CRISPR) technology is its use in creating genetic models of human disease. CRISPR technology can be used on induced pluripotent stem cells (iPSC) isolated from normal individuals to study a disease phenotype, or on IPSCs derived from disease patients to revert putative disease-causing mutations back to wild type^1,2^. The relative robustness of the CRISPR approach compared to zinc finger nucleases (ZFNs) and transcriptional activator-like effector nucleases (TALENs) has made testing accessible on protein coding mutations as well as empirical data generated by genome-wide association studies and other non-coding mutations^3,4^. Despite numerous successes, gene editing in iPSCs is challenged by the fact that homology directed repair (HDR), the process by which exogenous donor DNA is used to repair CRISPR-induced double strand breaks, is less efficient in iPSCs than transformed cancer cell lines^5–8^.

To overcome low HDR rates, researchers have adopted several strategies such as including antibiotic resistance genes on the CRISPR plasmid and/or donor DNA^9^ which, while effective, leaves an undesired insertion of foreign DNA into the genome. Combining positive selection markers with technologies that allow for excision of the selectable marker, such as the Cre/lox system or the footprint-free PiggyBAC transposon, represent notable improvements but extend timelines as clonal selection becomes a two-step process^2,10^. Methods that employ single-stranded oligo DNA nucleotide (ssODN) donor molecules avoid the issues that larger, double-stranded DNA molecules present with respect to random integration and unwanted ‘footprints’, but again are subject to the relatively low frequency of successful repair and sequence conversion around the site of the double-stranded break^7,11^. To contend with the difficulty of isolating rare clones, Miyaoka and colleagues devised a strategy using droplet digital PCR, pools of clones, and sib selection to enrich for extremely rare clones^12^. Additional strategies to increase the rate of HDR include timing the delivery of the Cas9 RNP complex to the nuclease by inducing cell cycle synchronization with known chemical inhibitors of cell cycle progression^13^. Here, while notable increases in HDR, up to 38%, can be achieved with synchronized HEK293 cells, synchronization had minimal effect in human primary fibroblasts or H9 human embryonic stem cells. Specifics of the ssODN structure and composition have also been shown to affect HDR rates. Lin *et al*. found that oligos with homology arms of at least 60 nucleotides were most effective, but that strand complementarity was not a factor. A more detailed investigation into how the structure of donor oligos affects HDR in HEK293 cells by Richardson *et al*. used insights gained from *in vitro* binding studies of Cas9 RNP-dsDNA complex. Using a GFP-reporter assay, they demonstrated that asymmetric donor oligos that are shorter with respect to the PAM site and are complementary to the (+) strand (i.e. non-target strand) were more effective in promoting HDR than symmetric donor oligos. A study optimizing HDR^14^ in iPSCs by Paquet *et al*. showed that efficient insertion of an intended mutation could be achieved with oligos that where complementary to the target strand (−) and that the frequency of the intended mutation integration was distance-dependent from the CRISPR cut site. Fidelity of HDR could also be increased by the introduction of silent base changes into the oligo that disrupted the CRISPR recognition sequence^15^.

We have carried out a systematic evaluation of the gene-editing steps in iPSCs to determine the best combination of delivery, CRISPR modality, and donor oligo design. We then tested the effects of a moderate ‘cold shock’ on the cells’ ability to carry out HDR. Our optimized method can successfully introduce desired genetic alterations into the genome with 10–30% efficiency through our novel combination of lipids designed for large RNA molecule delivery in conjunction with Cas9-encoding mRNA, symmetric donor oligos complementary to the target strand (−), and silent changes to prevent re-editing. Additional exposure of cells to a brief ‘cold shock’ of 32 °C can increase the amount of perfect HDR as much as two- to ten-fold in instances where low efficiency repair is observed at 37 °C.

## Results

### Efficient HDR in iPSCs using CRISPR/Cas9 RNA modality and lipid delivery

**Figure 1:**
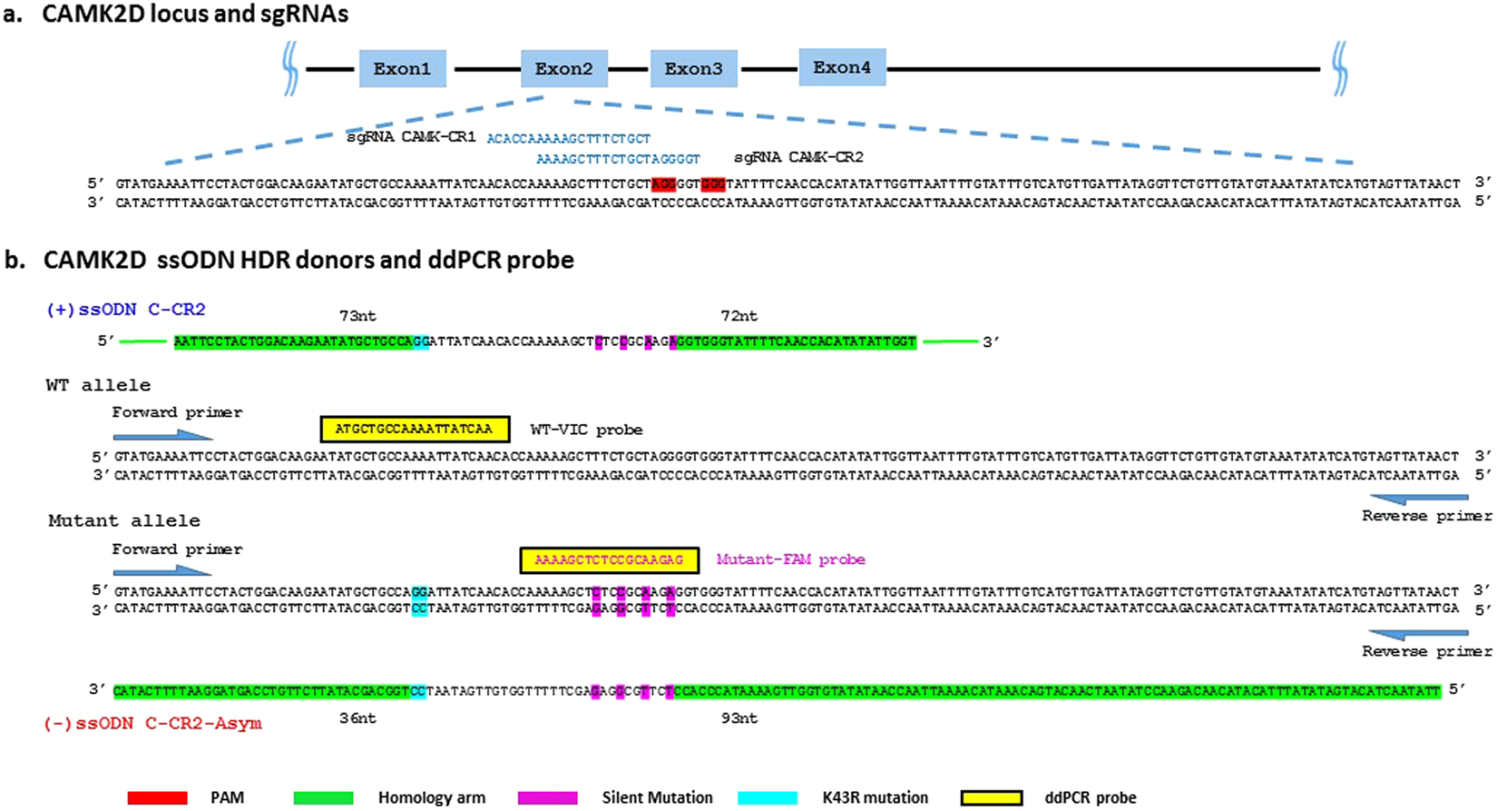
Single-stranded oligonucleotide (ssODN) donor design, droplet digital PCR probes and primer designs for gene editing and mutation detection at CAMK2D locus. (**a**) Two guide RNAs (CAMK-CR1 and CAMK-CR2) were designed to specifically target CAMK2D Exon2, CAMK-CR1 and CAMK-CR2 overlap by 14 nucleotides and were designed to cleave the DNA to introduce the same sequence alteration by HDR. (**b**) Two ssODN HDR donors (C-CR2 and C-CR2-Asym) were designed to introduce a kinase dead K43R mutation (AAA to AGG) and four silent mutations into Exon2 of the CAMK2D locus. The ssODN donor C-CR2 is a (+) strand HDR donor which is complementary to the guide RNA targeted cleavage strand with balanced homology arms around each side of the intended mutations (5′-73nt and 3′-72nt, respectively). C-CR2-Asym is a (−) strand HDR donor which is complementary to the guide RNA non-targeted strand with homology arms that differ in length (5′-93nt and 3′-36nt, respectively). To prevent subsequent re-cleavage, both donor oligo C-CR2 and C-CR2-Asym introduces three silent mutations within the guide CAMK-CR1 recognition site and one silent mutation within the PAM site. C-CR2 and C-CR2-Asym introduces four silent mutations within the guide CAMK-CR2 recognition site. A pair of primers and allele-specific probes conjugated with Vic or Fam fluorophores were also designed to detect separately the unaltered wild type alleles and mutated sequence conversion events. The forward primer was designed to anneal within the donor sequence while the reverse primer was designed to anneal outside of the donor sequence to ensure the proper locus was amplified.

**Figure 2:**
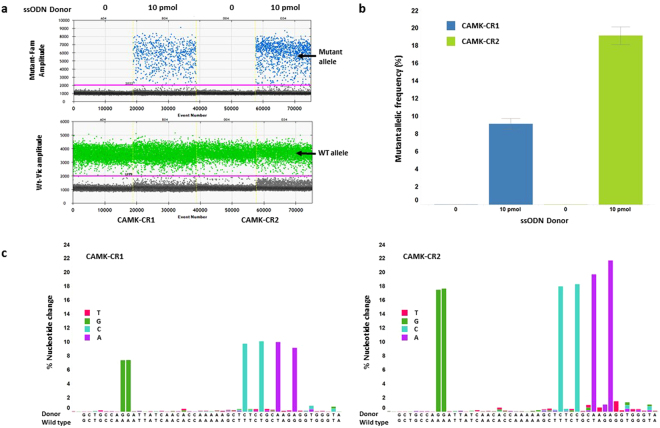
An optimized method for co-delivery of a single-stranded oligonucleotide (ssODN) donor and sgRNA/Cas9 mRNA to perform HDR at the CAMK2D locus in mc-iPSCs. sgRNA CAMK-CR1 or CAMK-CR2, Cas9 mRNA and ssODN donor C-CR2 were co-transfected into mc-iPSCs using the EditPro™ RNA transfection reagent (**a**) The percent wild type and mutant alleles from the transfected cells were detected by droplet digital PCR (ddPCR) using a wild type alleles specific fluorescence probes (VIC) and mutant alleles specific fluorescence probes (FAM), the fluorescence intensity of each droplet in the sample is plotted versus droplet number. Droplets that have fluorescence intensity above the pink threshold line are counted as positive for the target alleles. The bottom panel (green) represents the droplet of wild type alleles while the top panel (blue) represents the alleles that have undergone HDR. The data presented is from one representative experiment using the two sgRNAs at the best concentration of ssOND, 10 pmol. (**b**) Quantification of mutant allelic frequency. Data presented as mean ± SEM from four independent experiments. sgRNA CAMK-CR2 consistently produced HDR at greater than 15% of the total alleles. (**c**) Quantification of the intended nucleotide changes by next generation sequencing using the same sgRNA and donor used for the ddPCR experiments. Each bar represents one of the six nucleotide changes included on the donor oligo. The data indicates that complete sequence conversion occurred across the targeted region and at frequencies that were in agreement with the ddPCR results. The two A to G changes not directly measured by ddPCR were also incorporated albeit at lower frequencies compared to those changes that were closer to the CRISPR cleavage site. The data presented is the mean percent intended base alteration at each of their exact genomic coordinates from four independent experiments.

We evaluated several aspects of gene editing protocols in order to find the best conditions for generating HDR within the CAMK2D gene. First, we determined which CRISPR modalities [e.g. all-in-one plasmid DNA, sgRNA and Cas9 mRNA, or sgRNA *in vitro* transcription (IVT)/Cas9 ribronucleoprotein] and delivery methods (e.g. nucleofection or lipids formulated for enhanced delivery of large DNA and RNA molecules) generated the greatest the number of double-stranded breaks at two specific locations within the CAMK2D gene (Fig. 1a) as detected by PCR amplicon next-generation sequencing (NGS). The best NHEJ-induced indel rate for each modality and delivery method are presented in the supplementary data (Supplementary Table 1). the complete matrix of conditions used to determine optimal indel formation were then re-tested to determine the best combination of modality and delivery to promote HDR. We used a multiplexed ddPCR assay to measure the incorporation of four base changes designed to disrupt the CRISPR recognition sequence and, by proxy, the incorporation of the specific mutations designed to create a kinase-dead version of CAMK2D on the same oligo (Fig. 1b). The amount of wild type unedited sequence was determined using a different probe that specifically detected the non-HDR wild type alleles (Fig. 1b). The donor oligo design was symmetric with respect to the lengths of the homology arms and the CRISPR cleavage sites were located as close as possible to the intended kinase dead mutations. The donor sequence was also homologous to the non-targeting CRISPR cut strand (+). The four silent mutations introduced by the oligo altered the CRISPR recognition sequence for guide CAMK-CR2 in four positions and in guide CAMK-CR1, mutated the PAM sequence and introduced 3 sequence changes. The assay was validated using both synthesized fragments of the different DNA sequences, and on clones previously made in HEK293 cells known to be heterozygous and homozygous for the HDR donor oligo sequence (data not shown). The best HDR rate for all comparisons are present in Supplementary Table 1, and the data for the best combination presented in Fig. 2. For, IVT sgRNA/Cas9 mRNA and EditPro^TM^ lipid, we observed 9% of all alleles incorporating the donor oligo sequence for CAMK-CR1, and 19% of the alleles for CAMK-CR2 (Fig. 2b). To confirm the ddPCR results, we performed NGS on PCR amplicons from the transfected populations of iPSCs (Fig. 2c). For IVT sgRNA/Cas9 mRNA with both CAMK-CR1 and CAMK-CR2, the intended base changes that would indicate successful HDR into the locus were observed at their precise genomic coordinates and at frequencies that closely matched those determined by ddPCR, including the two guanine substitutions that were not directly measured by the ddPCR assay. These two substitutions, which are more distal to the CRISPR cut sites than the silent changes designed to disrupt sgRNA annealing, were observed at lower frequencies.

### ‘Cold shock’ increases the rate of HDR

Based on previous observations that a T-antigen temperature-sensitive immortalized cell line grown and maintained at 32 °C underwent HDR more efficiently than a similar cell line grown at 37 °C (data not shown), we tested if exposing the mc-iPS cell line to various 32 °C intervals would have an effect on the efficiency of HDR. The design of the experiment and the resulting percentages of the total alleles having undergone HDR as measured by ddPCR at each temperature are presented in Table 1. Using normal culturing conditions at 37 °C as the baseline for HDR (Group PL1), HDR frequencies of 7.50% and 5.0% for guide CAMK-CR1, and 16.16% and 8.86% for guide CAMK-CR2 at the 10 pmol and 30 pmol concentrations were observed respectively. When the cells were transferred to 32 °C immediately after transfection and kept at that condition for 24 hours, then moved to 37 °C for an additional 24 hours (Group PL2) we observed a statistically significant increase of between 1.8 to 2.3 fold in HDR. The effect was more pronounced at the 30 pmol concentration where lower HDR efficiencies were observed at baseline. Exposing the cells to 32 °C for 48 hour post transfection (Group PL3) also had a statistically significant effect on HDR, increasing it from 2.0 to 3.6 fold, with again the effect being more pronounced in conditions where HDR was lower at baseline.

**Table 1:** HDR efficiencies at various temperatures at the CAMK2D locus in mc-iPSCs as determined by ddPCR.

Group		PL1		PL2		PL3	
Conditions		37 °C-37 °C-37 °C		37 °C-32 °C-37 °C		37 °C-32 °C-32 °C	
sgRNA		CAMK-CR1	CAMK-CR2	CAMK-CR1	CAMK-CR2	CAMK-CR1	CAMK-CR2
ssODN input	None	0.02 ± 0.00	0.01 ± 0.00	0.02 ± 0.00	0.19 ± 0.07	0.00 ± 0.00	0.02 ± 0.00
	10 pmol	7.50 ± 0.54^a^	16.16 ± 0.76^c^	15.85 ± 0.51^a^	29.40 ± 0.81^c^	19.46 ± 0.47^a^	33.24 ± 1.53^c^
	30 pmol	5.02 ± 0.37^b^	8.86 ± 1.27^d^	13.79 ± 0.40^b^	20.70 ± 1.44^d^	18.14 ± 0.49^b^	27.16 ± 1.63^d^

The experiments were carried out at different temperatures as described in “material and methods” and HDR efficiencies were determined by ddPCR using probes specific for wild type and mutant sequences at the CAMK2D locus. Data presented as mean percentage of droplets containing the mutant allele ± standard error from three independent experiments and eight biological replicates.The significance of HDR efficiency difference among the three temperature conditions for each sgRNA and ssODN treatment were analyzed by one way ANOVA (a,b,c,d: one way ANOVA P < 0.0001, follow-up Dunnett’s multiple comparison, PL2 versus PL1: P = 0.0001, PL3 versus PL1: P = 0.0001).

### ‘Cold shock’ and alternate single-stranded oligo nucleotide donor designs affect the efficiency of HDR

**Figure 3:**
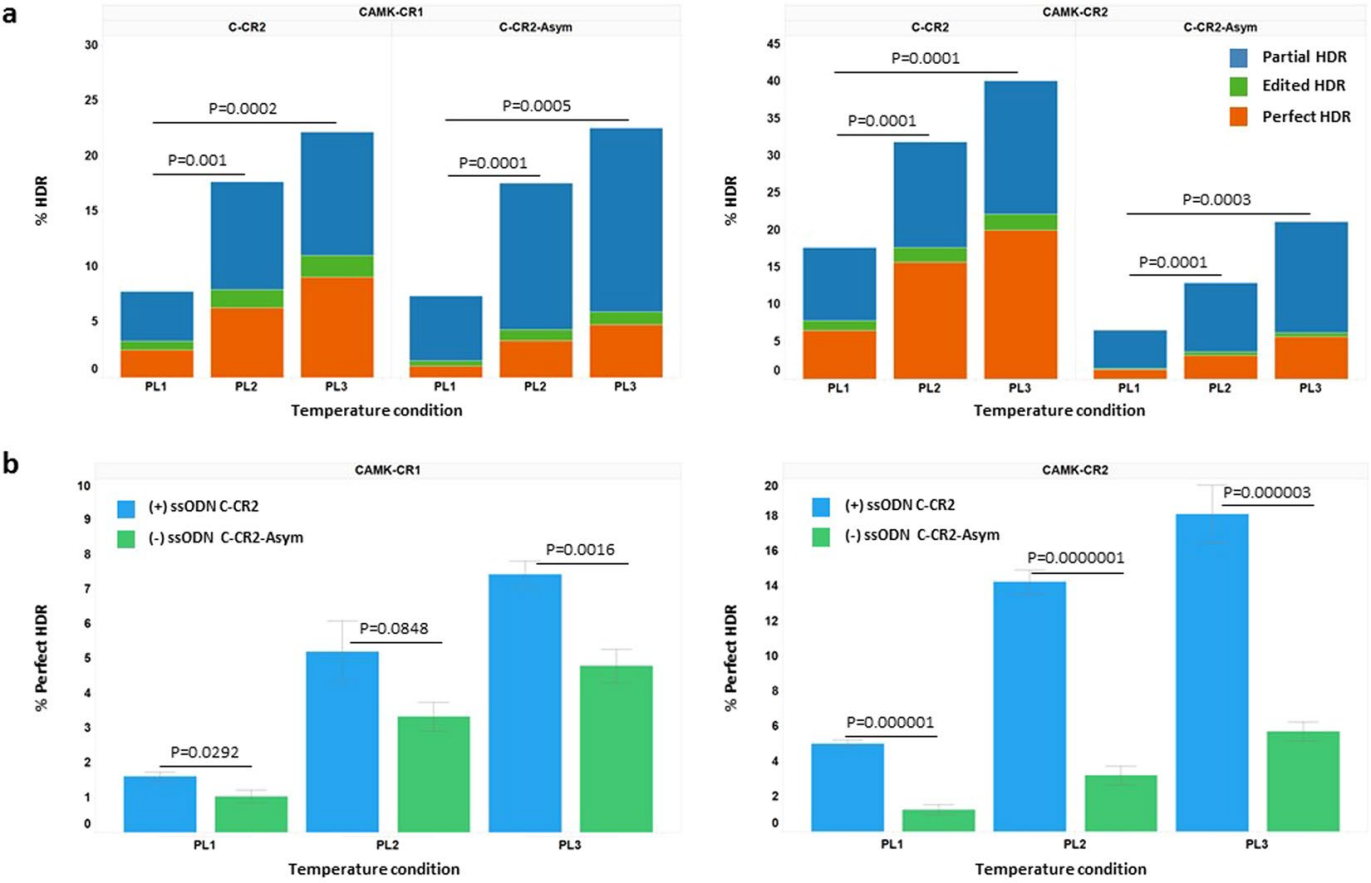
Effects of ‘cold shock’ and ssODN HDR donor designs on HDR efficiencies at the CAMK2D locus in mc-iPSCs as determined by NGS. Various amounts of ssODN C-CR2 or C-CR2-Asym were delivered to mc-iPSCs along with Cas9 mRNA and sgRNA CAMK-CR1 or CAMK-CR2 to achieve HDR at the CAMK2D locus. The experiments were carried out at different temperatures over 24 hour intervals as described in “material and methods”: PL1: 37 °C-37 °C-37 °C, PL2: 37 °C-32 °C-37 °C, PL3: 37 °C-32 °C-32 °C. (**a**) HDR events using 10 pmol of ssODN HDR donors for each treatment (CAMK-CR1 with C-TR2 or C-TR2-Asym, CAMK-CR2 with C-TR2 or C-TR2-Asym) were determined by NGS as described in “material and methods”. The data presented are the mean percent HDR events (C-CR2: 8 biological replicates from three independent experiments; C-CR2-Asym: 6 biological replicates from two independent experiments). The HDR types were categorized into three groups based on the resulting sequence around the region of the intended mutations. ‘Perfect HDR’: All intended base changes are present with no re-editing indels. Edited HDR: One or more of the intended base changes are present with re-editing indels present. Partial HDR: Some but not all of the intended base changes with no indels. Data demonstrates that increased HDR can be achieved by ‘cold shocking’ the cells and that the majority of the increase is in the ‘Perfect HDR’ category. The significance of total HDR efficiencies difference among the three temperature conditions for each sgRNA and ssODN treatment were analyzed by one way ANOVA (one way ANOVA: P < 0.0001 for all sgRNA and ssODN treatment, P value of follow-up Dunnett’s multiple comparison are shown in the figures). *HDR from 30 pmol ssODN and no oligo treatment are shown in the* Supplementary Table 3 (**b**) ‘perfect HDR’ events of each treatment (CAMK-CR1 with C-TR2 or C-TR2-Asym, CAMK-CR2 with C-TR2 or C-TR2-Asym) were plotted to compare ‘perfect HDR’ frequencies between the two ssODN designs. Data presented are the mean percent of ‘perfect HDR’ events ± SEM (6 biological replicates from two independent experiments), The difference of ‘perfect HDR’ frequencies between the two ssODN design in each treatment group were evaluated by Student’s T-test and p values are shown in the figures. (+) strand ssODN C-CR2 promote more ‘perfect HDR’ than (−) strand ssODN C-CR2-Asym across all temperature conditions.

Recent data suggest that precise donor oligo design can have dramatic effects on the efficiency of donor oligo HDR. More specifically, oligos that are asymmetric in length with respect to the CRISPR cut site (shorter on the side proximal to the cut site) and with sequence complementarity to the non-targeted strand (the strand not initially cleaved by Cas9) are more efficient promoters of HDR than the design we employed for editing the CAMK2D locus i.e., symmetric around the CRISPR cut site and complementary to the targeted strand^14^. To compare the two designs directly and further test the effects of cold shock on HDR, we designed a gene editing experiment whereby we compared the amount of HDR observed with the symmetrical targeted strand oligo donor to that observed with an asymmetrical non-targeted strand oligo donor designed to introduce the same sequence alternations (Fig. 1b). We determined the amount of HDR by amplicon based NGS and analyzed the resulting sequence data in several ways^16^:1) the amount of total HDR at the locus, i.e. oligo directed repair regardless of whether all or part of the intended changes are present; 2) the percentage HDR that represented ‘perfect HDR’, oligo directed repair with all six intended base changes intact; 3) the percentage of HDR for which the sequence that had putatively undergone re-editing once repaired by virtue of indels being re-introduced into the converted sequence; and 4) the percentage of HDR where partial oligo-directed repair occurred such that sequence is missing the two more distal sequence changes (the intended CAMK2 kinase dead mutations (Fig. 3a and Supplementary Table 3).

As initially observed by ddPCR, guide CAMK-CR2 was more efficient in promoting total HDR than guide CAMK-CR1 at baseline conditions, i.e., cell transfected and maintained at 37 °C, albeit at lower overall HDR (Figs 2b and 3a). The amount of total HDR across all temperature conditions and oligo concentrations were also comparable for guide CAMK-CR1. In general, statistically significant increases in total HDR were observed across all comparisons of temperature, guide and oligo design. For guide CAMK-CR1 the amount of total HDR for both oligos, across the three temperature conditions was essentially the same and where an approximately 2.9 fold increase in total HDR was observed under the PL3 temperature condition for both oligo designs (Fig. 3a). For CAMK-CR2, the fold increase in total HDR for the symmetrical oligo, C-CR2 was approximately 2.4 fold but the magnitude of the response was greater than that observed for guide CAMK-CR1, where 40% of the alleles had undergone some type of HDR. For the asymmetric design, C-CR2-Asym, a total increase of HDR of 3.5 fold was observed between PL1 and PL3 however the magnitude of the response was approximately half of that observed with the symmetrical guide (Fig. 3a). However when only considering the amount of ‘perfect’ HDR observed as a result of ‘cold shock’, the type of oligo used had a dramatic effect especially for guide CAMK-CR2 where the differential in total HDR was higher between the two designs to begin with and where symmetrical oligo design was superior in directing conversion of all six nucleotide changes (Fig. 3b). For guide CAMK-CR1, where the amount of total HDR was similar for both oligo types across all temperature conditions, the amount of ‘perfect’ HDR was again greater for the symmetrical oligo however the difference was only statistically significant under the PL3 conditions (Fig. 3b). The percentage of the total alleles with indels for both guides, oligo type and temperature condition is presented in Supplementary Fig. 1. As observed by others^17^ ‘cold shock’ produced an increase in overall indel formation in the absence of any repair oligo but the effects were far less than the overall increase in total HDR. As expected, the percent indel formation drops with the addition of repair oligo as the locus is converted back to ‘wild type’ minus the intended sequence alterations and is commensurate with the amount of total HDR observed under each condition (Supplementary Fig. 1a,b)

**Figure 4:**
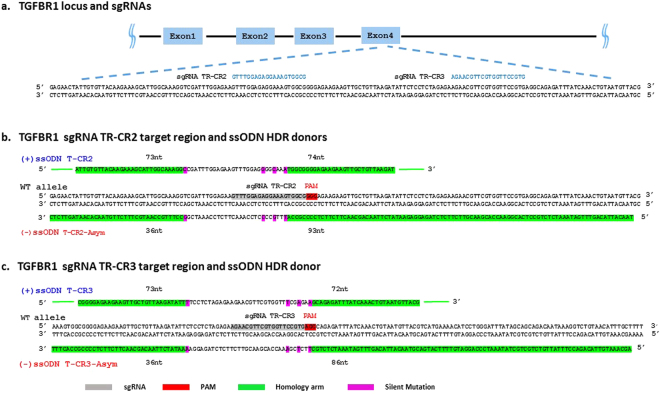
Guide RNAs and single-stranded oligonucleotide (ssODN) donor designs for gene editing at the TGFBR1 locus. (**a**) Two guide RNAs TR-CR2 and TR-CR3 were designed to specifically target TGFBR1 Exon4 and introduce different sequence alterations by HDR. TR-CR3 is 39 nucleotides downstream of TR-CR2. (**b**) Two ssODN HDR donors (T-CR2 and T-CR2-Asym) were designed to introduce a silent mutation 12 bp upstream of guide RNA TR-CR2 target site, with three silent mutations within the guide RNA recognition sequence to prevent re-editing of the HDR converted sequence. T-CR2 is a (+) strand HDR donor which is complementary to the guide RNA targeted cleavage strand with balanced homology arms around each side of the intended mutations (5′-73nt and 3′-74nt, respectively). T-CR2-Asym is a (−) strand HDR donor which is complementary to the guide RNA non-targeted strand with homology arms that differ in length (5′-93nt and 3′- 36nt, respectively). (**c**) Two ssODN donors (T-CR3 and T-CR3-Asym) were designed to introduce a known SNP 12 bp upstream of guide RNA TR-CR3 target site, with two silent mutations within the guide RNA recognition sequence and one silent mutation within the TR-CR3 PAM site to prevent re-editing of the HDR converted sequence. T-CR3 is a (+) strand HDR donor which is complementary to the guide RNA targeted strand with balanced length homology arms (5′-73nt and 3′-72nt, respectively) around each side of the intended mutations. T-CR3-Asym is a (−) strand HDR donor which is complementary to the guide RNA non-targeted strand with unbalanced length homology arms (5′-86nt and 3′-36nt, respectively) around each side of the intended mutations.

**Figure 5:**
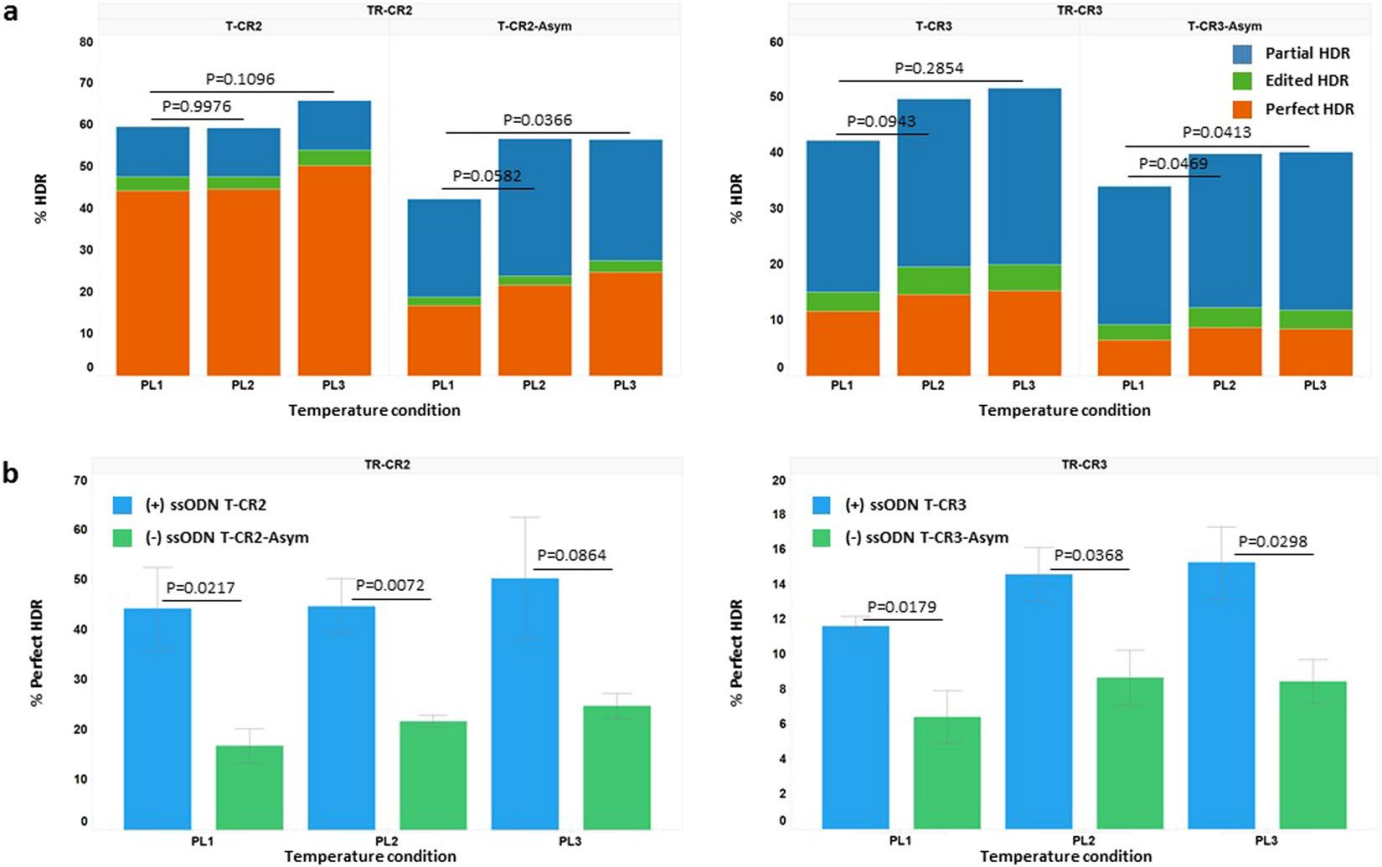
Effects of ‘cold shock’ and ssODN HDR donor designs on HDR efficiency at the TGFBR1 locus in mc-iPSCs. ssODN HDR donors and sgRNAs were co-delivered into mc-iPSCs cells along with Cas9 mRNA to achieve HDR at the TGFBR1 locus. Experiments were carried out at different temperatures over 24 hour intervals as described in “material and methods”: PL1: 37 °C-37 °C-37 °C, PL2: 37 °C-32 °C-37 °C, PL3: 37 °C-32 °C-32 °C. (**a**) HDR events using 10 pmol of ssODN HDR donors for each treatment (TR-CR2 with T-TR2 or T-TR2-Asym, TR-CR3 with T-TR3 or T-TR3-Asym) were determined by NGS as described in “material and methods”. The data presented are the mean percentage HDR events (4 biological replicates from three independent experiments). The HDR types were categorized into three groups based on the resulting sequence around the region of the intended mutations. ‘Perfect HDR’: All intended base changes are present with no re-editing indels. Edited HDR: One or more of the intended base changes are present with re-editing indels. Partial HDR: Some but not all of the intended base changes with no indels. The significance of total HDR efficiencies differences among the three temperature conditions for each sgRNA and ssODN treatment were analyzed by one way ANOVA (one way ANOVA: TR-CR2 with T-TR2, P = 0.4037; TR-CR2 with T-TR2-Asym, P = 0.0208; TR-CR3 with T-TR3, P = 0.1846; TR-CR3 with T-TR3-Asym, P = 0.0139; P value of follow-up Dunnett’s multiple comparison are shown in the figures). HDR from 30 pmol ssODN and no oligo treatment are shown in the Supplementary Table 4 (**b**) events using 10 pmol of ssODN HDR donors for each treatment (TR-CR2 with T-TR2 or T-TR2-Asym, TR-CR3 with T-TR3 or T-TR3-Asym) were plotted to compare ‘perfect HDR’ frequencies between the two ssODN designs. Data presented are the mean percent of ‘perfect HDR’ events ± SEM (4 biological replicates from three independent experiments). The difference of ‘perfect HDR’ frequencies between the two ssODN designs in each treatment group were evaluated by Student’s T-test and p values are shown in the figures. Across all temperature conditions, (+) ssODN strand T-CR2 and T-CR3 promote more ‘perfect HDR’ than (−) ssODN strand T-CR2-Asym and T-CR3-Asym, respectively.

To extend these observations to another locus and to further test the effects of ‘cold shock’ and oligo design on HDR, we designed a gene editing experiment to insert silent changes and SNPs into the TGFRB1 locus. The locations of the two guides tested are shown in Fig. 4a and the sequences of the four donor oligos, the positions of the intended sequence alterations and their relationship to the guide positions are shown in Fig. 4b,c. Guide TR-CR2, was designed to cut 30 bps 3′ to the intended T to C sequence change at genomic coordinate 9:101900208. Both symmetric and asymmetric donor oligos also contained 3 additional silent sequence changes designed to disrupt guide recognition and re-editing at the locus. The length of the homology arms are also listed in Fig. 4b. Guide TR-CR3 was designed to direct cleavage 31 bps 3′ from the intended C to T sequence change at genomic coordinate 9:101900268, a known high frequency SNP (rs202156059) in the TGFBR1 gene chosen for its putatively innocuous nature (Fig. 4c). As with TR-CR2 and its ssODNs, three additional silent sequence alterations were also included to prevent re-editing of the converted locus. The length of the homology arms were designed to be as close as possible to the ssODNs used for guide RNA TR-CR2 (Fig. 4c) Using the IVT sgRNA/Cas9 RNA lipid format, both CRISPRs were efficient at generating indels in the mc-iPSC line, where, in the absence of repair oligos, the percent of alleles with indels as determined by NGS were 94% for TR-CR2 and 58% for TR-CR3, (Supplementary Fig. 2a,b). In the presence of both repair oligos, guide TR-CR2 lead to very efficient total HDR rates of 60% for the symmetric oligo T-CR2 and 42% for the asymmetric design at condition PL1, 37 °C (Fig. 5a and Supplementary Table 4). For guide TR-CR3, total HDR percentages of 41 and 34 were observed at 37 °C for the symmetrical and asymmetrical designs respectively (Fig. 5a).

As observed with CAMK2D, culturing the cells at 32 °C for either 24 hours (Group PL2) or 48 hours (Group PL3) produced an increase in HDR; however, the effect was generally minimal given the relativity high rates of HDR to begin with at 37 °C and for the most part did not reach statistical significance (Fig. 5a). However, the ‘cold shock’ effect was more pronounced for the asymmetric oligos where the amount of ‘perfect HDR’ at 37 °C was less than the symmetric oligos. Here statistical significance was achieved for guide TR-CR2 and asymmetric donor T-CR2-Asym when the amount of total HDR observed at 37 °C (PL1) was compared to the amount observed when the cells were culture at 32 °C for 48 hours (PL3) and for guide TR-CR3 and the asymmetric donor T-CR3-Asym at both the PL2 and PL3 conditions. However when only considering the amount of ‘perfect’ HDR, observed as a result of ‘cold shock’ as well as at baseline conditions, the type of oligo used had a dramatic effect. Across all comparison but one (TR-CR2/PL3), the symmetrical donor oligos were statistically significantly superior at directing ‘perfect’ HDR repair than their asymmetric counter parts (Fig. 5b).

The rate of indel formation for guide TR-CR2 and TR-CR3 in the absence (previously mentioned above) and presence of donor oligo are presented in Supplementary Fig. 2a,b. In the absence of oligo, the percentage of total alleles with indels for guide TR-CR2 at 37 °C was greater than 90%. Here, ‘cold shock’ had no measurable effect on increasing percentage indel formation. In the presence of oligo, the percent indels dropped in accordance with the amount of total HDR observed at the locus (Fig. 5a). For guide TR-CR3, the percent indels at 37 °C was approximately 60%. Here, ‘cold shock’ did have a measurable effect of indel formation increasing the percentage approximately by 20% (Supplementary Fig. 2b).

### ‘Cold shock’ is more effective when base HDR rates are lower

To test whether ‘cold shock’ was effective in a cell type other than the particular mc-iPSC line under investigation, we repeated the identical CAMK2D gene editing experiment used to derive the data in Table 1 in HEK293 cells and determined the levels HDR using ddPCR (Supplementary Table 5) and specific HDR categories by NGS (Supplementary Fig. 3a and Supplementary Table 6). Baseline total HDR levels as determined by ddPCR for both CAMK2D sgRNAs and two donor oligo concentrations were approximately 1% at 37 °C (Supplementary Table 5). These results were obtained using both plasmid based all-in-one CRISPR modality (data not shown) as well as the IVT sgRNA/Cas9RNA modality used for iPSCs in independent experiments. This is in contrast to that observed in the mc-iPSC line where total HDR levels were over 7% and 17% respectively for CAMK-CR1 and CAMK-CR2 at the 10 pmol concentration (Fig. 3a). Transfection condition for iPSC may not be optimal for HEK293 and that may partially contribute to the lower HDR in the HEK293 cell lines. The degree by which the locus and the cell type can determine the levels of gene editing have been described by others^8^. However, despite the low HDR rates observed at 37 °C, ‘cold shock’ under both sets of conditions, 24 hours (PL2) and 48 hours (PL3), produced for the best sgRNA, CAMK-CR2, a statistically significant 6.9-fold increase in total HDR at both the 24 hour and 48 hour conditions as determined by ddPCR (Supplementary Table 5). Both guides and all conditions produced statistically significant increases in total HDR in response to ‘cold shock’ (Supplementary Table 5). Amplicon based NGS and analysis confirmed that ‘cold shock’ produced statistically significant increase in total HDR for both guides with the exception of the PL1 vs PL3 comparison for guide CAMK-CR2. In general, increases in ‘perfect HDR’ exceeded 10 fold for both guides and all conditions (Supplementary Fig. 3a and Supplementary Table 6).

The amount of NHEJ-associated indel formation with each temperature condition and treatment is presented in Supplementary Fig. 3b. ‘Cold shock’ produced an approximately 2.4 fold increase in NHEJ in the 24 hour condition (PL2) and a 2 fold increase in the 48 hour condition (PL3) for guide CAMK2D-CR1 and a 5.5 fold and 4 fold increase for guide CAMK2D-CR2 under the same conditions. These larger effects are driven by the fact that guide CAMK2D-CR2 is consistently less potent in producing NHEJ indels at 37 °C (Supplementary Figs 1b and 3b). In the presence of donor oligo, the percentage of indels in the population decreases as the double-stranded breaks undergo oligo directed repair; however, the percentage of indel formation continues to decrease in the 30 pmol treatments even though the rates of HDR are lower that at the 10 pmol concentration.

## Discussion

The rapid development of CRISPR-based genome engineering methodologies requires an agnostic and systematic process of evaluation in order to gain the maximum benefit from this technology. Here we report an optimized CRISPR modality/delivery combination that is highly effective in promoting HDR in the mc-iPSC line. We then used this method to evaluate if exposure to lower temperature can increase the efficiency of HDR and found that exposure 32 °C or ‘cold shock’ for 24 or 48 hours is capable of increasing rates of HDR 2 fold or more. Given that appreciable effort is being exerted to find ways of increasing rates of HDR, including ‘driving’ the repair process away from non-homologous end joining toward HDR with chemical inhibition of DNA repair enzymes^18^ and blocking and synchronizing cells at the G2/M boundary with other inhibitors^13^, our method provides a more ‘physiological’ approach that might have broader application, especially where gene editing is being applied in a therapeutic setting. Interestingly, the ‘cold shock’ effect is more dramatic when lower HDR rates are observed (1–20% of the alleles) and diminishes as the base HDR rate increases above 30% or more. In fact, the largest increase in total HDR fold change was in with HEK293 cells where the baseline HDR at 37 °C was in the 1% range. The diminishing of the ‘cold shock’ response at high baseline HDR suggests a theoretical limit to the number of alleles that can be altered, at least with this approach. The exact mechanism by which the ‘cold shock’ increases HDR is currently under investigation. A mechanism similar to how zinc finger nucleases increase indel formation may be plausible^17^. We observe that with very efficient CRISPRs the effect of the ‘cold shock’ on indel formation is minimal, as observed with CAMK2D guide CAMK-CR1 and TGFBR1 guide TR-CR2. Conversely, when cutting efficiencies and indel formation are lower, as that observed at the CAMK2D locus in HEK293 cells, the increases in cutting efficiency and indel formation are more pronounced. Increased indel formation can clearly contribute to higher HDR rates but does not account for all the increase observed with ‘cold shock’ or explain why ‘perfect HDR’ can be favored under cold conditions (Fig. 3a). A possible mechanism that may contribute to increased HDR rates is that growing cells at 32 °C impacts the cell cycle, with more cells accumulating in G2/M; however, our initial observations to date do not show any cell cycle effects to support this hypothesis (data not shown). A third and more likely contributing factor is that the cold has a thermodynamic effect that acts to stabilize recombination intermediates. Work is currently ongoing to understand the mechanism in detail. One potential concern is that the ‘cold shock’ might adversely affect pluripotency. Preliminary analysis looking at three standard markers of pluripotency, Oct4, SSEA3, and Nanog^19,20^ suggest this is not an issue, although some loss of Nanog expression may occur with prolonged exposure to cold (Supplementary Fig. 4). Clearly more work is needed to ensure that ‘cold shock’ is effective and generalizable across cell lines and applications.

Clearly, another important component and consideration for obtaining efficient HDR is the structure of ssODN. Although many potential designs can be envisioned, we have tested what many considered to be the standard design, symmetrical length homology arms with strand complementary to the target strand to an alternative design that employs asymmetric homology arms, with the short arm being proximal to the CRISPR cut site and strand complementary to the non-targeted strand^14^. Across the two loci tested, both oligo designs were fairly equivalent in their ability to promote total HDR to high levels when used in combination with efficient guides and an efficient and optimized lipid delivery system. Both oligo designs also responded to ‘cold shock’ with increases in total HDR in a nearly equivalent fashion, although the symmetrical design tended to work better. Where the oligo designs differentiated themselves was in how they could promote HDR over a greater distance from the CRISPR cut site to promote sequence alterations, or ‘perfect HDR’. This difference was significant at 37 °C but more importantly the increase in ‘perfect’ HDR can represent the majority of the increase in HDR as the result of ‘cold shock’(Fig. 3a,b). These data suggest that if the desired genomic sequence alteration or multiple changes are required across the length of the donor oligonucleotide, that symmetrical targeted strand oligos, in conjunction with ‘cold shock’ might represent the preferred approach. While these data are in disagreement with that of Richardson *et al*.^14^, our data do agree with those of Paquet *et al*., whose donor oligos were designed to the same strand^15^.

In summary, we have developed a protocol for performing gene editing in iPSCs that does not require the use of nucleofection or selection to obtain a population of cells that have efficiently undergone directed genomic sequence alteration by the process of HDR. We have also shown that HDR can be effectively increased by the incorporation of a simple, brief, and physiological exposure to lower temperature which will have broad utility across many genome engineering applications.

## Materials and Methods

### Cell lines and cell culture

Human mc-iPS cells^21^ were obtained from System Biosciences (SC301A-1) and were maintained on Matrigel (BD Bioscience) coated plates in mTeSR media (Stem Cell Technologies) with 50 units/ml penicillin-streptomycin (Thermo Fisher Scientific) and with daily medium changes^22^. For passaging, the cells were washed with PBS and treated with Accutase (Thermo Fisher Scientific) at 37 °C°C for 5 min. The cells were re-suspended in mTeSR media, centrifuged at 80 g for 5 min and cell pellets re-plated in mTeSR media supplemented with 10 μM ROCK Inhibitor Y-27632 (Cayman Chemical).

### CRISPR and Cas9 Reagents

CRISPR guide RNAs were designed using the Doench algorithm (http://portals.broadinstitute.org/gpp/public/) and Zhang laboratory CRISPR design tool (http://crispr.mit.edu). The guide sequences were either subcloned into plasmid pX458 (GenScript) or synthesized as IVT sgRNAs (Thermo Fisher Scientific). GeneArt™ Platinum™ Cas9 Nuclease was obtained from Thermo Fisher Scientific and Cas9 mRNA (5meC, Ψ) was obtained from TriLink BioTechnologies. The repair templates (Ultramer, IDT) were designed as single-stranded oligodeoxynucleotides (ssODN) with target mutations to the middle of the oligonucleotide with homologous genomic flanking sequence on the both side of mutations^8,14^. In some ssODN designs, silent mutations were also introduced within the guide RNA recognition sequences and PAM sites. PCR primers were designed using PRIMER 3 and primers were purchased from Sigma. Sequences of the primers, probes, and oligonucleotide donors used in the study are listed in Supplementary Table 2.

### Transfections

For lipid based transfection of mc-iPSC with IVT sgRNA/ Cas9 mRNA, 1 × 10^5^ cells were plated into each well of a Matrigel coated 24 well plate in mTeSR media one day prior to the transfection. On the day of transfection, the media was first replaced with 0.5 ml of fresh media, 480 ng of IVT sgRNA and 2 µg of Cas9 mRNA were then mixed in 50 µl of OptiMEM medium in a Eppendorf tube and followed by adding 2.5 µl of mRNA-In Stem® or EditPro^TM^ (MTI-GlobalStem) per tube. The mixture was incubated at room temperature for 15 min and the entire mixture was then added to the well drop by drop. For homology directed repair experiments, ssODNs were re-suspended in water at 10 µM and various amounts added to the complex before lipid addition. 100 ng of GFP mRNA was also spiked into each mixture to monitor the transfection efficiency. The plates were incubated at 37 °C in a 5% CO_2_ incubator and the cells were then harvested for genomic DNA extraction 48 h later. Additional transfection methods in iPSC and HEK293 are in supplementary methods.

### Genomic DNA extraction and PCR amplification of edited regions

iPS Cells were harvested by gently aspirating the media from each well and treating the cells with 250 µl of Accutase (Thermo Fisher Scientific) at 37 °C for 10 min. 750 µl of mTeSR media was then added to each well and the cell suspension was transferred to a 1.5 ml Eppendorf tubes and spun at 1000 g for 5 min. Genomic DNA was then extracted using DNeasy Blood & Tissue Kit (QIAGEN) and 100 ng of genomic DNA used for PCR using Q5 polymerse (NEB) and target specific primers (Supplementary Table S2). Specifically, PCR amplification of the CAMK2D locus was done using primer CAMK2D-F and CAMK2D-R. PCR amplification of TGFBR1 locus was done using primer TGFBR1-F and TGFBR1-R. Amplification was carried out at 98 °C for 30 s, 31 cycles of 98 °C 10 s, 63 °C 30 s, 72 °C 1 min, followed by 1 min final extension at 72 °C, and cooled to 4 °C.

### Next generation sequencing and analysis

Sequencing libraries were generated from PCR amplicons as follows: After the PCR step high molecular weight (HMW) template DNA and residual PCR primers were removed in a two-step cleanup process. HMW DNA was first removed by adding 0.6 v/v ratio Ampure XP beads (Beckman Coulter) and transferring the cleared supernatants to a new 96 well plate. Primers were then removed by adding 1.2 v/v Ampure XP beads to the transferred supernatants and placed on a magnet again until clear. The supernatants were then discarded and the beads washed 2 × with 80% ethanol, air dried, and resuspended in 20 µl H_2_O to elute the DNA. DNA quality was assessed using a Tapestation HSD5000 (Agilent Technologies) and quantified with a Qubit HS DNA (Invitrogen). The purified PCR products were used as input for the Nextera XT kit (Illumina) using manufacturer’s recommendations. Samples were uniquely indexed with up to 384 unique i5/i7 combinations using Illumina standard indexing kits. Amplification was carried out at 72 °C for 3 min, 98 °C for 1 min, then 12–14 cycles of 98 °C 30 s, 55 °C 30 s, 72 °C 1 min, followed by 5 min final extension at 72 °C, and cooled to 4 °C. Libraries were size selected according to the same Ampure XP bead protocol as described previously, and eluted in 15 µl H_2_O. Eluted products were run on Tapestation HSD1000 (Agilent) and quantified by qPCR using KAPA Library (Kapa Biosystems) Quantification kit and an ABI Vii7. Individual library concentrations were calculated using the Kapa Library Quantification template. Each library was diluted to 4 nM and pooled. The final pool was denatured and diluted to 12pM following Illumina’s standard protocol using 1% v/v PhiX as a spiked in control. Run parameters were set at 150 bp paired-end, dual indexed 8 bp each using a MiSeq. 300v2 reagent kit (Illumina). Samples were demultiplexed using MiSeq Reporter v2.6 or bcl2fastq v2.17.

A bioinformatics pipeline was developed to analyze the NGS data to determine indel and HDR frequencies^16^. Quality filtering was performed on paired-end reads using PRINSEQ. Reads with quality score mean below 30 or length shorter than 50 were removed. The remaining reads were then aligned to a reference genome with BWA, followed by realignment using ABRA (Assembly-Based ReAligner) to enhance detection of large indels^23^. We examined the read coverage for each amplicon, and surveyed the whole amplicon region for insertion and deletion frequencies. To assess CRISPR efficiency, we defined a simple measurement, % indel reads, which represents the percentage of reads that have CRISPR-induced indels among all reads that span the window of sgRNA sequence (about 20 bases without PAM). For a read to be considered an indel read, it must have at least one inserted or deleted base inside this window, whereas a wild-type read must have no indels within the window. To evaluate homology-directed repair (HDR) effectiveness, we examined point mutation frequencies in the window and its flanking regions, and categorized the sequences into four groups: (1) ‘Perfect HDR’: the repair has all intended base changes with no indels between them; (2) Edited HDR: some or all intended base changes occur, but indels are present, likely due to re-editing by CRISPR after the directed repair; (3) Partial HDR: some but not all intended base changes occur, with no indels within the repaired sequence. This could be caused by partial repair or fragmentation of DNA strand during the NGS procedure.

### ddPCR assay to detect CAMK2D wild type and mutation sequences

The QX200^TM^ Droplet Digital PCR System (Bio-Rad laboratories, CA) was used as suggested by the manufacturer. ddPCR assays for detecting CAMK2D wild-type and mutated sequences were designed using Primer Express and ordered from Life Technologies (Life Technologies, CA, USA). ddPCR reactions were assembled using standard protocols: ddPCR Super mix for Probes (no dUTP) (Bio-Rad laboratories, CA, USA) was combined with 160ng of sample genomic DNA, 1 µl of 20 × FAM assay and 1 µl of 20 × VIC assay (1 × CAMK2D-ddPCR primer F & CAMK2D-ddPCR primer R at 900 nM each, 1 × probes at 250 nM each), 5 units of restriction enzyme BamHI –HF® (New England BioLabs, MA), and water for a final reaction volume of 20 µl. Reactions were converted into approximately 20,000 one-nanoliter droplets using the QX200 Droplet Generator and transferred to a 96-well plate for thermal cycling per manufacturer recommendation. After thermal cycling, droplets were read on the QX200 Droplet Reader and assigned as positive or negative based on fluorescence amplitude. The primer and probe sequences are listed in Supplementary Table S2.

### ‘Cold shock’ experiment on transfected cells

One day prior to transfection, mc-IPSCs were seeded in 24 well plates as described in the transfection section and divided into four groups (PL1-PL3). Group PL1 to group PL3 were incubated at 37 °C for 24 h. The cells were then transfected with IVT sgRNA/Cas9 mRNA and ssODN using EditPro^TM^ as described. After transfection, group PL1 was kept at 37 °C until harvested 48 hr later while PL2 was transferred to 32 °C for 24 h then returned to 37 °C for the final 24 h. Group PL3 was transferred to 32 °C after transfection and kept at this temperature until harvested 48 h later. The cells were harvested for genomic DNA isolation 48 h post transfection as described and indel formation or HDR were determined by either ddPCR or NGS.

### Statistical analysis

One-way ANOVA with Dunnett’s post test was performed using GraphPad Prism 7 (GraphPad Software) to compare the HDR efficiencies among three temperature conditions for each sgRNA and ssODN treatment. Two-tailed Student’s t-test was performed using GraphPad Prism 7 to compare the difference of ‘perfect’ HDR between symmetric and asymmetric donor oligo design, a P-value ≤ 0.05 was used to define statistically significant differences.

## Electronic supplementary material

Supplementary Information
