# Supplementary material for: ‘Cold shock’ increases the frequency of homology directed repair gene editing in induced pluripotent stem cells
Source: Sci Rep. 2018 Feb 1;8:2080. doi: 10.1038/s41598-018-20358-5 (PMC5794742; doi:10.1038/s41598-018-20358-5)
Supplement: Supplementary file 1 — Supplementary Information [file 41598_2018_20358_MOESM1_ESM.pdf]

Supplementary information

'Cold shock' increases the frequency of homology directed repair gene editing in induced pluripotent stem cells

Q. Guo, G. Mintier, M. Ma-Edmonds, D.Storton, X. Wang, X. Xiao, B. Kienzle, D. Zhao, and John N. Feder*

Emerging Technologies and Genomics

Bristol-Myers Squibb Co.

Pennington, NJ 08534, USA

* Corresponding author

John.Feder@bms.com

Table of Contents

Supplemental Figures and Tables

Supplementary Figure 1. Effects of ‘cold shock’ and ssODN HDR donor on Indel efficiency at the CAMK2D locus in mc-iPSCs.

Supplementary Figure 2. Effects of ‘cold shock’ and ssODN HDR donor designs on Indel efficiency at the TGFBR1 locus in mc-iPSCs.

Supplementary Figure 3. ‘Cold shock’ enhances HDR and indel efficiencies at the CAMK2D locus in HEK293T cells.

Supplementary Figure 4. Expression of pluripotency markers in mc-iPSCs after ‘cold shock’

Supplementary Table 1. Best Indel and HDR rates for different delivery methods and CRISPR modalities at the CAMK2D locus

Supplementary Table 2. sgRNAs and oligonucleotides used in this study

Supplementary Table 3. Effects of ‘cold shock’ and ssODN HDR donor design on HDR efficiency at the CAMK2D locus in mc-iPSCs as determined by NGS

Supplementary Table 4. Effects of ‘cold shock’ and ssODN HDR donor design on HDR efficiency at the TGFBR1 locus in mc-iPSCs as determined by NGS

Supplementary Table 5. HDR efficiencies at various temperatures at the CAMK2D locus in HEK293T cells as determined by ddPCR

Supplementary Table 6. ‘Cold shock’ enhances HDR efficiency at the CAMK2D locus in HEK293T cells as determined by NGS

Supplementary Figure 1. Effects of 'cold shock' and ssODN HDR donor on Indel efficiency at the CAMK2D locus in mc-iPSCs.

a. **Indels introduced by CAMK-CR1 at the CAMK2D locus in mc-iPSCs as detected by next generation sequencing**

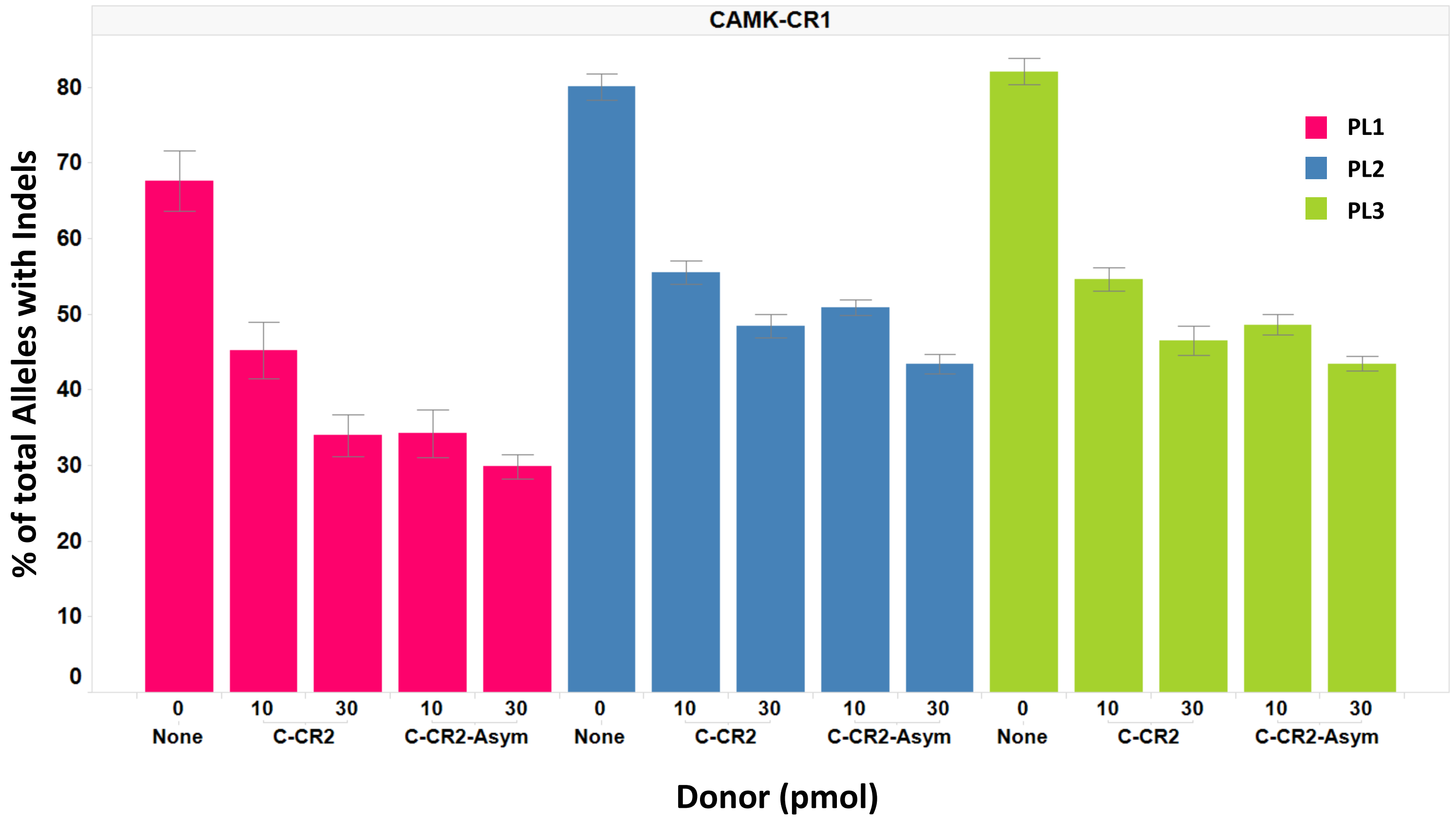

b. Indels introduced by CAMK-CR2 at the CAMK2D locus in mc-iPSCs as detected by next generation sequencing

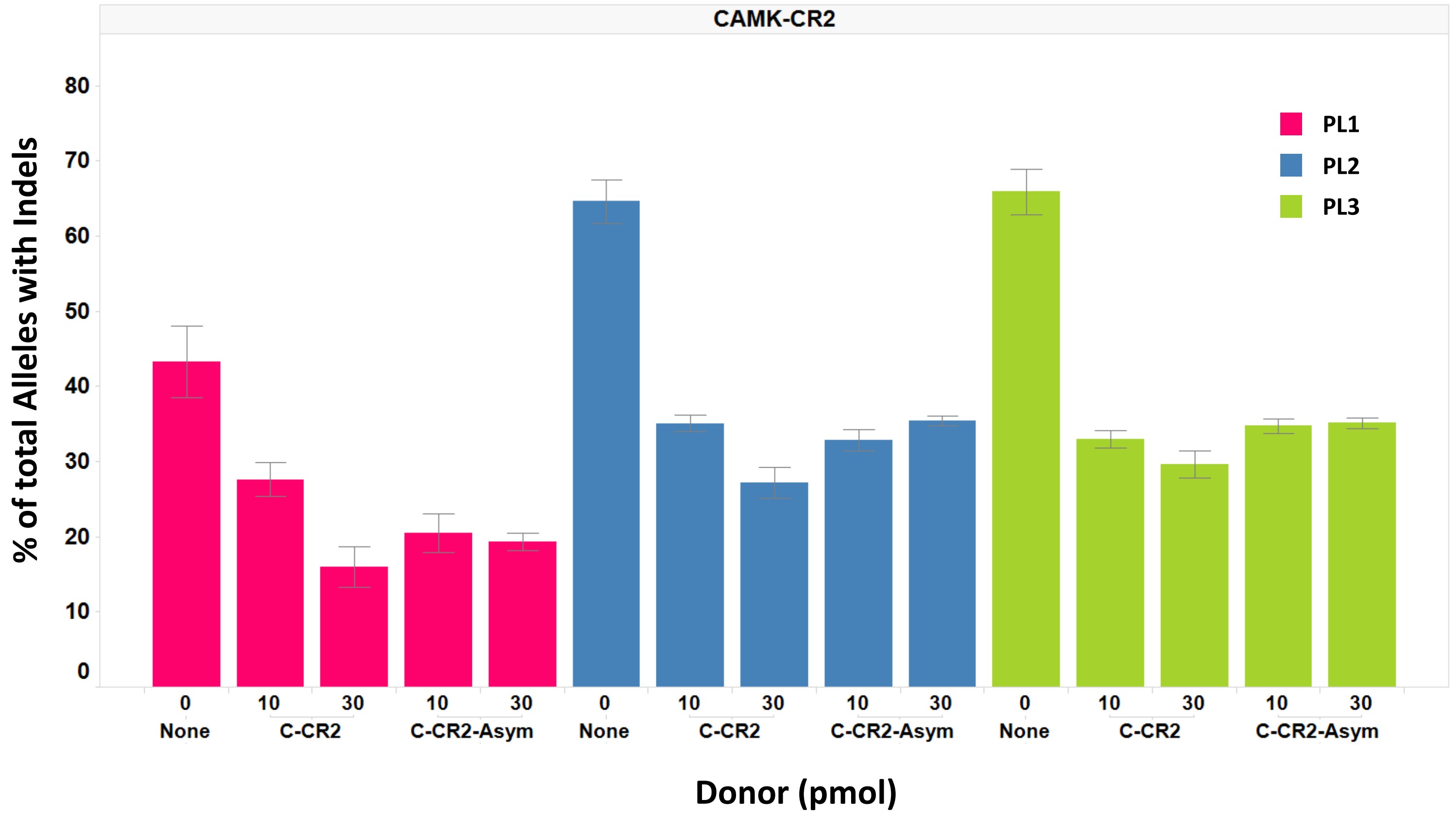

Supplementary Figure 1. Effects of 'cold shock' and ssODN HDR donor on Indel efficiency at the CAMK2D locus in iPSC cells as determined by NGS. Various amounts of ssODN C-CR2 or C-CR2-Asym were delivered to mc-iPSCs along with Cas9 mRNA and sgRNA CAMK-CR1 or CAMK-CR2 to achieve HDR at the CAMK2D locus. The experiments were carried out at different temperatures over 24 hour intervals as described in "material and methods": PL1: 37°C-37°C-37°C, PL2: 37°C-32°C-37°C, PL3: 37°C-32°C-32°C. The percentage of the total alleles incurring indels in the absence and the presence of the ssODN across all temperature conditions were determined by NGS as described in "material and methods". The data presented are mean percentage of indel ±SEM (C-CR2: 8 biological replicates from three independent experiments; C-CR2-Asym: 6 biological replicates from two independent experiments). (a) Indel introduced by sgRNA CAMK-CR1 with or without donor C-CR2 and C-CR2-Asym, (b) Indel introduced by sgRNA CAMK-CR2 with or without donor C-CR2 and C-CR2-Asym. Data demonstrates that both guides effectively introduced double stranded break into the CAMK2D locus in the absence of oligo as revealed by the amount of NHEJ indel formation and that the 'cold shock' could produce additional increases in the percent of total alleles with indels especially for guide CAMK-CR2 whose efficiency in creating indels was less than guide CMAK-CR1. The percent of the alleles with indels decreases in the presence of oligo.

Supplementary Figure 2. Effects of 'cold shock' and ssODN HDR donor designs on Indel efficiency at the TGFBR1 locus in mc-iPSCs.

a. **Indels introduced by TR-CR2 at the TGFBR1 locus in mc-iPSCs as detected by next generation sequencing**

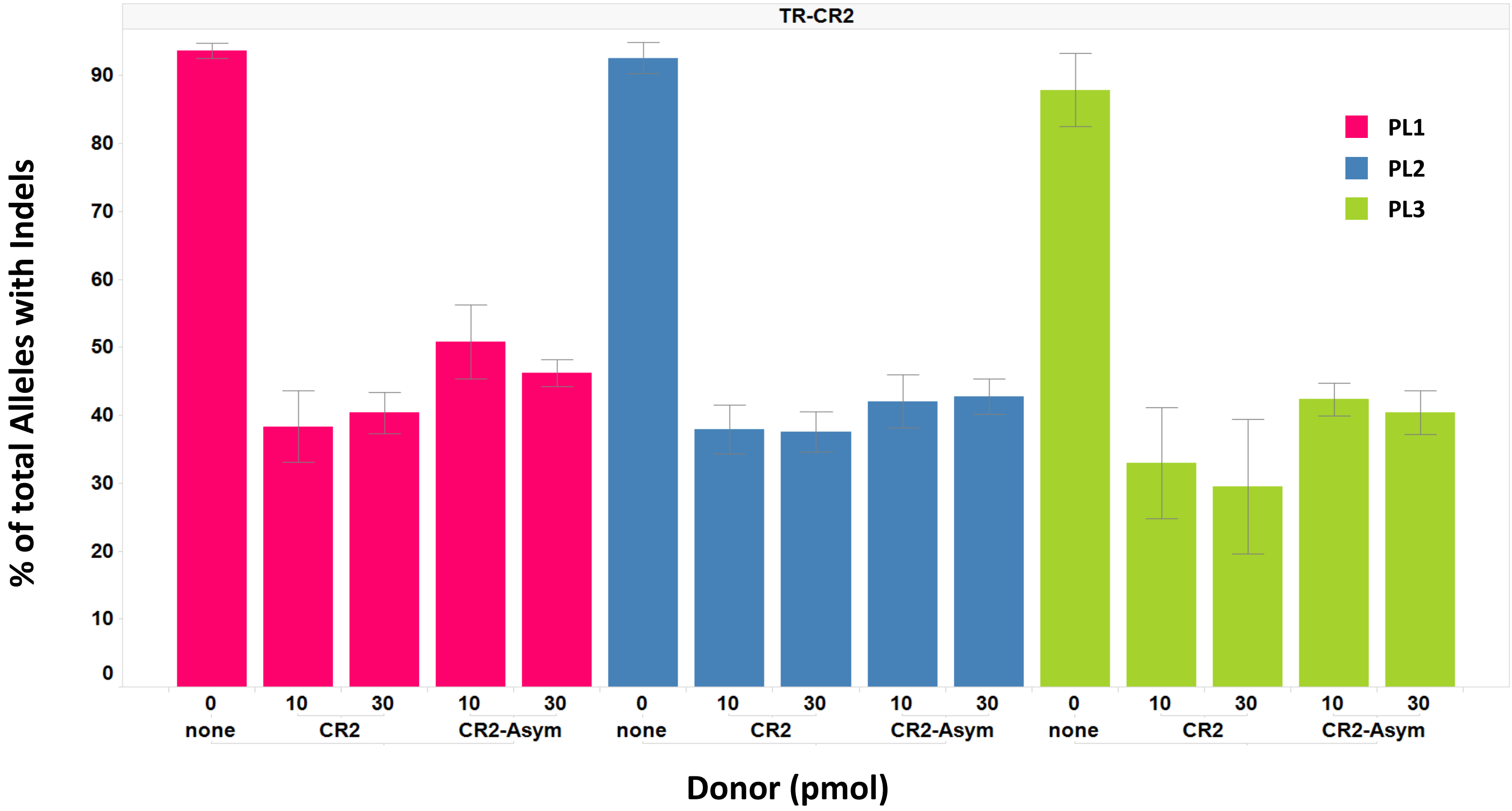

b. Indels introduced by TR-CR3 at the TGFBR1 locus in mc-iPSCs as detected by next generation sequencing

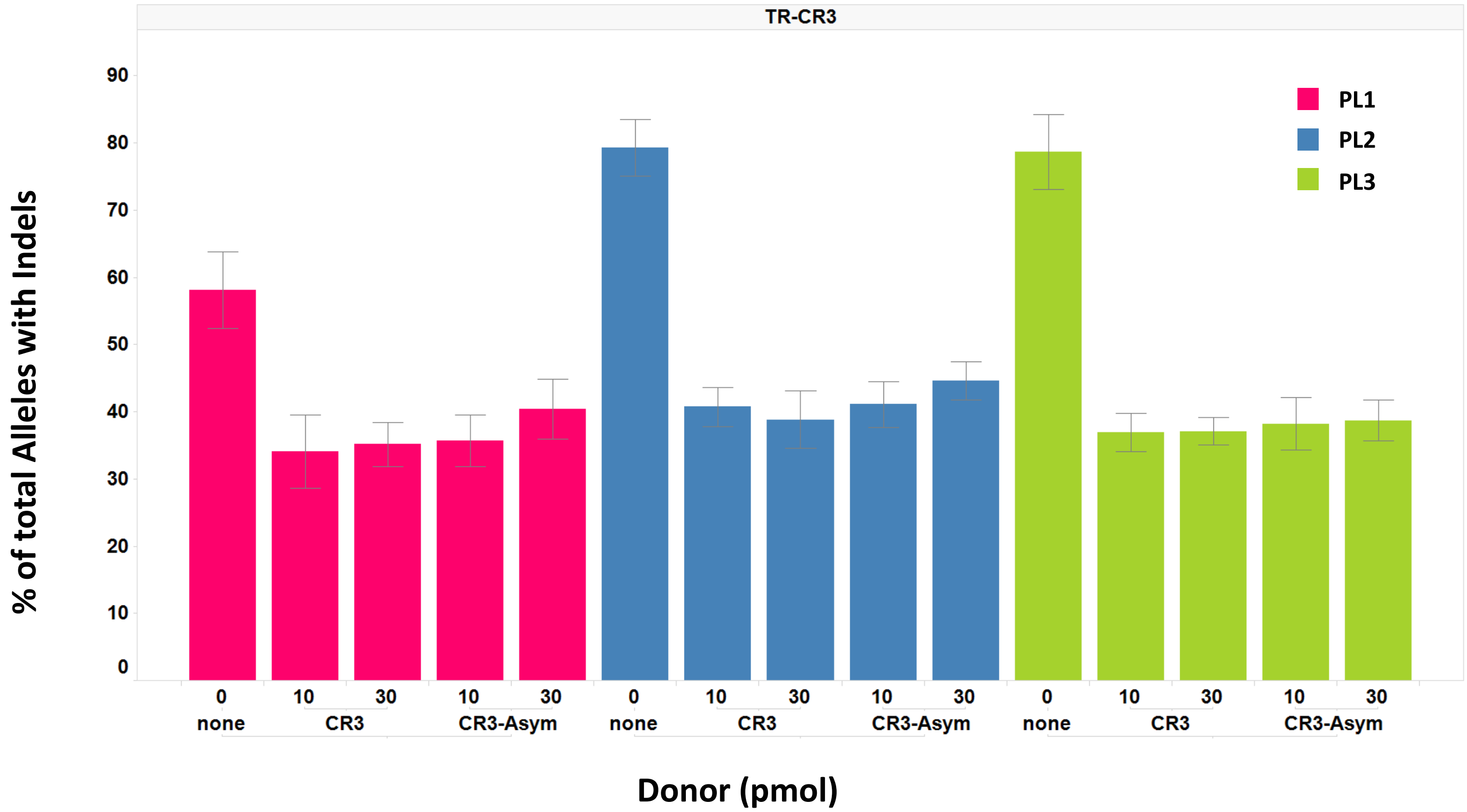

Supplementary Figure 2. Effects of 'cold shock' and ssODN HDR donor designs on Indel efficiency at the TGFBR1 locus in mc-iPSCs. ssODN HDR donors and sgRNAs were co-delivered into mc-iPSCs cells along with Cas9 mRNA to achieve HDR at the TGFBR1 locus. Experiments were carried out at different temperatures over 24 hour intervals as described in "material and methods": PL1: 37°C-37°C-37°C, PL2: 37°C-32°C-37°C, PL3: 37°C-32°C-32°C. The percentage of the total alleles incurring indel formation in the absence and presence of the ssODN across all temperature conditions were detected by NGS. (a) Indel introduced by sgRNA TR-CR2 with or without donor T-CR2 and T-CR2-Asym, (b) Indel introduced by sgRNA TR-CR3 with or without donor T-CR3 and T-CR3-Asym. The data presented are the mean percentage indels ±SEM (4 biological replicates from three independent experiments).

Supplementary Figure 3. 'Cold shock' enhances HDR and indel efficiencies at the CAMK2D locus in HEK293T cells.

a. **Categories of HDR at the CAMK2D locus in HEK293T cells as detected by next generation sequencing**

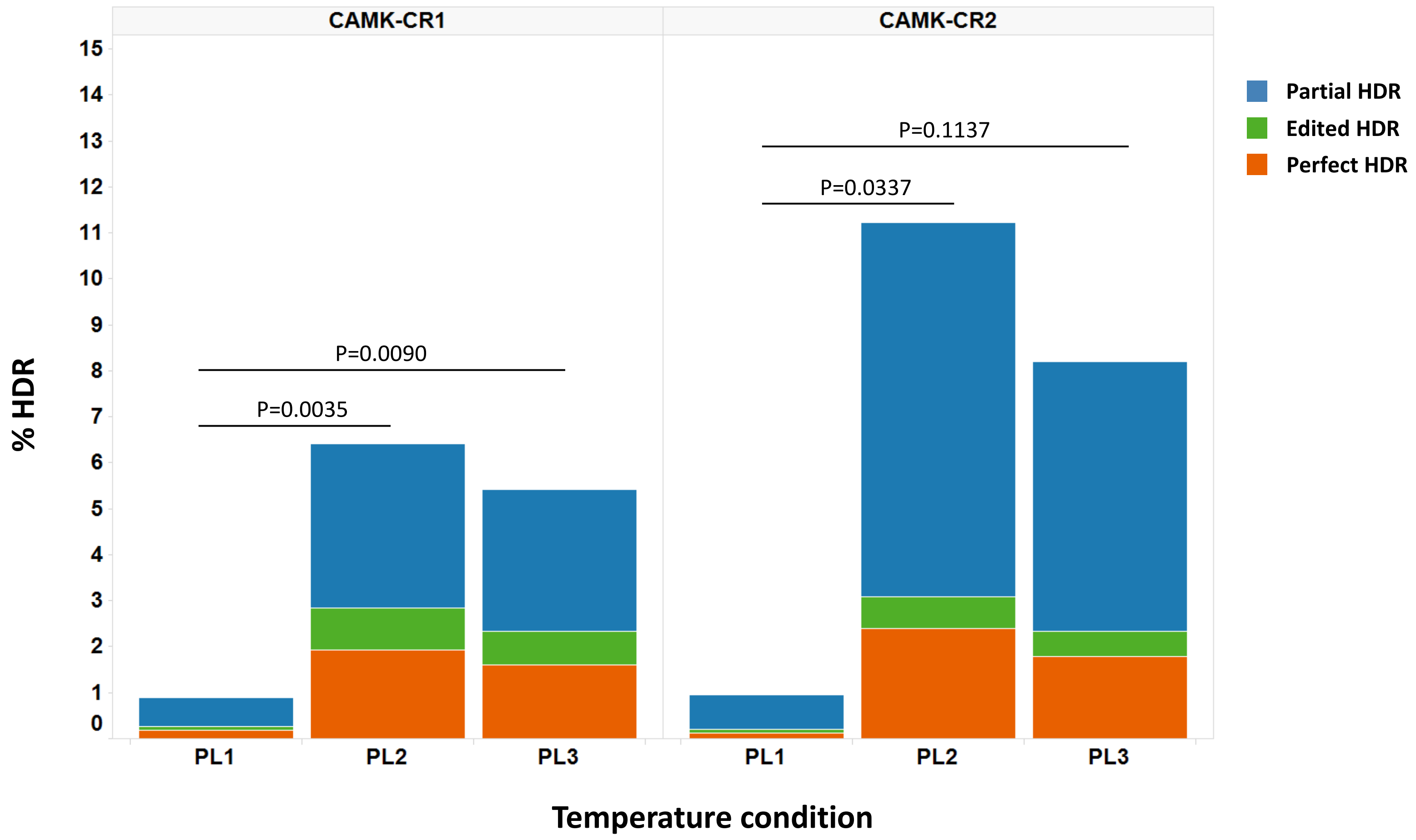

b. Indels at the CAMK2D locus in HEK293T cells as detected by next generation sequencing

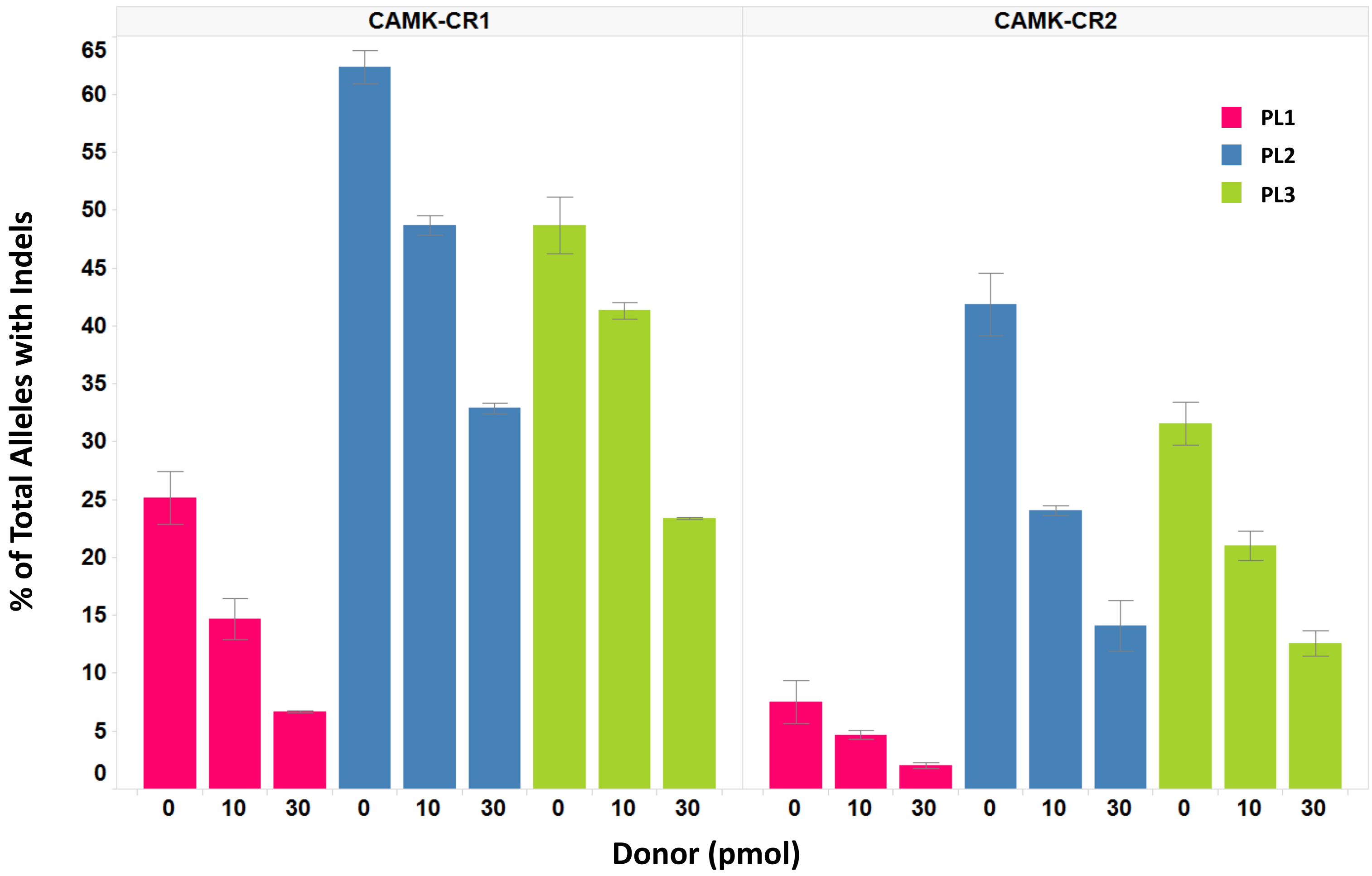

Supplementary Figure 3. ‘Cold shock’ enhances HDR and indel efficiencies at the CAMK2D locus in HEK293T cells. Various amount of ssODN C-CR2 were delivered to HEK293T cells along with Cas9 mRNA and sgRNA CAMK-CR1 or CAMK-CR2 using the same transfection conditions as for mc-iPSC to achieve HDR at the CAMK2D locus. The experiments were carried out at different temperatures over 24 hour intervals as described in “material and methods”: PL1: 37°C-37°C-37°C, PL2: 37°C-32°C-37°C, PL3: 37°C-32°C-32°C (a) HDR events using 10 pmol of ssODN HDR donor for each treatment were determined by NGS as described in “material and methods”. Data presented as the mean percentage HDR events from three biological replicates. The HDR types were categorized into three groups based on the resulting sequence around the region of intended mutations. **Perfect HDR**: All intended base changes occur and no indels. **Edited HDR**: One or more intended base changes occur, but there are indels. **Partial HDR**: Some but not all intended base changes occur, and no indels. The significance of total HDR efficiencies difference among three temperature conditions for each sgRNA and ssODN treatment were analyzed by one way ANOVA (one way ANOVA: CAMK-CR1 with C-CR2, P=0.0041; CAMK-CR2 with C-CR2, P=0.0469; P value of follow-up Dunnett’s multiple comparison are shown in the figures). *HDR from 30 pmol ssODN and no oligo treatment are shown in Supplementary table 6*. (b) The percentage of total alleles incurring indel formation in the absence and presence of the ssODN across all temperature conditions. Indels around the sgRNA target site for each treatment were determined by NGS. Data presented is the mean ± SEM percentage indel events from three biological replicates

Supplementary Figure 4. Expression of pluripotency markers in mc-iPSCs after ‘cold shock’

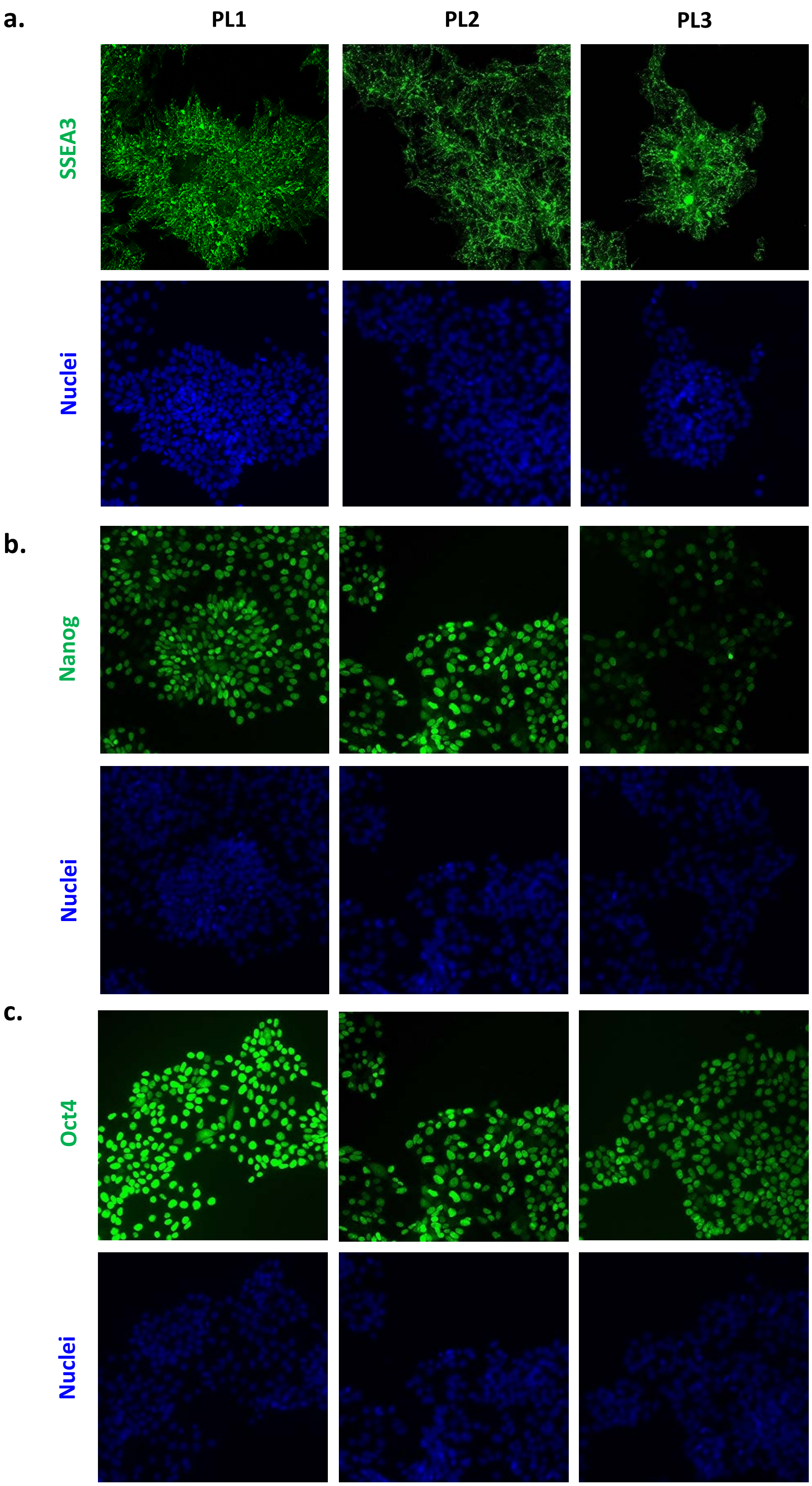

Supplementary Figure 4. Expression of pluripotency markers in mc-iPSCs after 'cold shock'. Mc-iPSC were grown at different temperatures over 24 hour intervals as described in "supplementary methods": PL1: 37°C-37°C-37°C, PL2: 37°C-32°C-37°C, PL3: 37°C-32°C-32°C. The cells were then stained with pluripotency specific antibodies as described in "supplementary methods": (a) SSEA3 (green), (b) Nanog (green) and (c) OCT4 (green). The cells were also co-stained with Hoechst to label nuclei (blue).

Supplementary Table 1. Best Indel and HDR rates for different delivery methods and CRISPR modalities at the CAMK2D locus

**Transfection methods**	**Nucleofection**			**Lipid**					
**sgRNA format**	**DNA format**	**RNA format**		**DNA format**		**RNA format**			
**Cas9/sgRNA source**	**All-in-one**	**sgRNA/Cas9 RNP**	**sgRNA/Cas9 mRNA**	**All-in-one**		**sgRNA/Cas9 RNP**		**sgRNA/Cas9 mRNA**	
**Transfection reagents or instrument**	**4D-Nucleofector™**			**DNA-In® Stem**	**DNA-In® CRISPR**	**mRNA-In® Stem**	**EditPro™**	**mRNA-In® Stem**	**EditPro™**
**Best Indel rate**	29.5%[b, f]	7.7%[a]	1.0%[a]	43.5%[a]	28.3%[a]	36.3%[a]	22.4%[a]	26.0%[a]	38.8%[a]
**Best HDR rate**	2.6%[c,d]	1.0%[c,d]	***	2.9%[c,d]	2.7[c,d]	3.2%[e]	4.5%[e]	9.3%[e]	26.1%[e]

*** Not tested due to very low Indel rate

a. sgRNA CAMK-CR1
b. sgRNA CAMK-CR2
c. sgRNA CAMK-CR1 and ssODN C-CR2
d. Only sgRNA CAMK-CR1 was tested in these experiments
e. sgRNA CAMK-CR2 and ssODN C-CR2
f. sgRNA CAMK-CR1 showed 25.8% Indel rate

Supplementary Table 2. sgRNAs and oligonucleotides used in this study

Primers used for genomic DNA PCR

Name	Target	Forward (5' to 3')	Reverse (5' to 3')
CAMK2D-F and CAMK2D-R	CAMK2D	TGGGTTTCCAGGAAGAATTG	TCCCTCTCAAAAGCAAAAGG
TGFBR1-F and TGFBR1-R	TGFBR1	GGTTTACCATTGCTTGTTCAGAG	TGCCCTAAACTAAACCAACAAA

Primers used for droplet digital PCR

Name	Target	Forward (5' to 3')	Reverse (5' to 3')
CAMK2D-ddPCR primer F & R	CAMK2D	GCATTCTCAGTGGTGAGAAGATGT	CAGACCAAGAAGCATTCAGGAA

Probes used for droplet digital PCR

Name	Target	Probe for wild type allele	Probe for mutant type allele
CAMK2D-ddPCR probe	CAMK2D	ATGCTGCCAAAATTATCAA	AAAAGCTCTCCGCAAGAG

sgRNAs sequences

Name	Target	Sequence (5' to 3')	PAM sequence
**CAMK-CR1**	CAMK2D	ACACCAAAAAGCTTTCTGCT	AGG
**CAMK-CR2**	CAMK2D	AAAAGCTTTCTGCTAGGGGT	GGG
**TR-CR2**	TGFBR1	GTTTGGAGAGGAAAGTGGCG	GGG
**TR-CR3**	TGFBR1	AGAACGTTCGTGGTTCCGTG	AGG

Supplementary Table 2 continued. sgRNAs and oligonucleotides used in this study

Single-stranded oligonucleotide used in the homology directed repair experiments

HR ssODN	Target	Sequence (5' to 3')
C-CR2	CAMK2D	TTGGTTTCCAGGGGGGCATTCTCAGTGGTGAGAAGATGTATGAAAATTCCTACTGGACAAGAATATGCTGCCAGGATTATCAA CACCAAAAAGCTCTCCGCAAGAGGTGGGTATTTTCAACCACATATATTGGTTAATTTTGTATTTGTCATGTTGATTATAGGTT CTGTTGTATGT
C-CR2-Asym	CAMK2D	TTATAACTACATGATATATTTACATACAACAGAACCTATAATCAACATGACAAATACAAAATTAACCAATATATGTGGTTGAA AATACCCACCTCTTGCGGAGAGCTTTTTGGTGTTGATAATCCTGGCAGCATATTCTTGTCCAGTAGGAATTTTCATAC
T-CR2	TGFBR1	CTTTAGGTTTACCATTGCTTGTTCAGAGAACAATTGCGAGAACTATTGTGTTACAAGAAAGCATTGGCAAAGGcCGATTTGGA GAAGTTTGGAGgGGcAAaTGGCGGGGAGAAGAAGTTGCTGTTAAGATATTCTCCTCTAGAGAAGAACGTTCGTGGTTCCGTGA GGCAGAGAT
T-CR2-Asym	TGFBR1	TAACATTACAGTTTGATAAATCTCTGCCTCACGGAACCACGAACGTTCTTCTCTAGAGGAGAATATCTTAACAGCAACTTCTT CTCCCCGCCATTTGCCCCTCCAAACTTCTCCAAATCGGCCTTTGCCAATGCTTTCTTGTAACACAATAGTTCTC
T-CR3	TGFBR1	GCATTGGCAAAGGTCGATTTGGAGAAGTTTGGAGAGGAAAGTGGCGGGGAGAAGAAGTTGCTGTTAAGATATTTTCCTCTAGA GAAGAACGTTCGTGGTTTCGAGAAGCAGAGATTTATCAAACTGTAATGTTACGTCATGAAAACATCCTGGGATTTATAGCAGC AGACAATAAAGGT
T-CR3-Asym	TGFBR1	AGCAAATGTTACAGACCTTTATTGTCTGCTGCTATAAATCCCAGGATGTTTTCATGACGTAACATTACAGTTTGATAAATCTC TGCTTCTCGAAACCACGAACGTTCTTCTCTAGAGGAAAATATCTTAACAGCAACTTCTTCTCCCCGCCACTTT

Supplementary Table 3. Effects of 'cold shock' and ssODN HDR donor design on HDR efficiency at the CAMK2D locus in mc-iPSCs as determined by NGS

	sgRNA	CAMK-CR1							
	ssODN	None							
	ssODN Input	None							
Group	Temperature	(PctPerfectOligo)		(PctEditedOligo)		(PctPartialOligo)		(PctNonOligo)	
		Average	StdErr	Average	StdErr	Average	StdErr	Average	StdErr
PL1	37°C-37°C-37°C	0	0	0.11	0.02	0.04	0.01	99.85	0.02
PL2	37°C-32°C-37°C	0	0	0.15	0.05	0.06	0.01	99.79	0.06
PL3	37°C-32°C-32°C	0.02	0.01	0.1	0.02	0.08	0.04	99.8	0.05

	sgRNA	CAMK-CR1							
	ssODN	C-CR2							
	ssODN Input	10 pmol							
Group	Temperature	(PctPerfectOligo)		(PctEditedOligo)		(PctPartialOligo)		(PctNonOligo)	
		Average	StdErr	Average	StdErr	Average	StdErr	Average	StdErr
PL1	37°C-37°C-37°C	2.47	0.58	0.82	0.13	4.48	0.53	92.23	1.2
PL2	37°C-32°C-37°C	6.32	0.98	1.65	0.18	9.75	0.65	82.29	1.75
PL3	37°C-32°C-32°C	9.09	1.12	1.96	0.16	11.15	0.51	77.8	1.29

	sgRNA	CAMK-CR1							
	ssODN	C-CR2							
	ssODN Input	30 pmol							
Group	Temperature	(PctPerfectOligo)		(PctEditedOligo)		(PctPartialOligo)		(PctNonOligo)	
		Average	StdErr	Average	StdErr	Average	StdErr	Average	StdErr
PL1	37°C-37°C-37°C	1.75	0.48	0.8	0.21	2.96	0.44	94.5	1.05
PL2	37°C-32°C-37°C	5.71	0.96	1.45	0.2	7.13	0.43	85.71	1.55
PL3	37°C-32°C-32°C	8.41	1.16	1.8	0.25	9.2	0.48	80.59	1.57

Supplementary Table 3 continued. Effects of 'cold shock' and ssODN HDR donor design on HDR efficiency at the CAMK2D locus in mc-iPSC as determined by NGS

	sgRNA	CAMK-CR1							
	ssODN	C-CR2-Asym							
	ssODN Input	10 pmol							
Group	Temperature	(PctPerfectOligo)		(PctEditedOligo)		(PctPartialOligo)		(PctNonOligo)	
		Average	StdErr	Average	StdErr	Average	StdErr	Average	StdErr
PL1	37°C-37°C-37°C	1.03	0.19	0.51	0.08	5.87	0.74	92.6	0.97
PL2	37°C-32°C-37°C	3.33	0.42	0.99	0.07	13.24	0.84	82.44	1.3
PL3	37°C-32°C-32°C	4.79	0.48	1.16	0.14	16.59	1.09	77.46	1.62

	sgRNA	CAMK-CR1							
	ssODN	C-CR2-Asym							
	ssODN Input	30 pmol							
Group	Temperature	(PctPerfectOligo)		(PctEditedOligo)		(PctPartialOligo)		(PctNonOligo)	
		Average	StdErr	Average	StdErr	Average	StdErr	Average	StdErr
PL1	37°C-37°C-37°C	0.89	0.16	0.46	0.06	5.28	0.52	93.37	0.71
PL2	37°C-32°C-37°C	3.33	0.37	0.9	0.04	13.22	0.9	82.56	1.28
PL3	37°C-32°C-32°C	5.53	0.29	1.37	0.15	17.08	1.08	76.01	1.39

Supplementary Table 3 continued. Effects of 'cold shock' and ssODN HDR donor design on HDR efficiency at the CAMK2D locus in mc-iPSC as determined by NGS

	sgRNA	CAMK-CR2							
	ssODN	None							
	ssODN Input	None							
Group	Temperature	(PctPerfectOligo)		(PctEditedOligo)		(PctPartialOligo)		(PctNonOligo)	
		Average	StdErr	Average	StdErr	Average	StdErr	Average	StdErr
PL1	37°C-37°C-37°C	0	0	0.2	0.05	0.28	0.07	99.52	0.07
PL2	37°C-32°C-37°C	0.02	0.01	0.47	0.12	0.28	0.06	99.23	0.17
PL3	37°C-32°C-32°C	0	0	0.52	0.05	0.33	0.08	99.15	0.1

	sgRNA	CAMK-CR2							
	ssODN	C-CR2							
	ssODN Input	10 pmol							
Group	Temperature	(PctPerfectOligo)		(PctEditedOligo)		(PctPartialOligo)		(PctNonOligo)	
		Average	StdErr	Average	StdErr	Average	StdErr	Average	StdErr
PL1	37°C-37°C-37°C	6.54	1.02	1.35	0.39	9.8	0.79	82.32	2.05
PL2	37°C-32°C-37°C	15.73	1.1	1.95	0.43	14.27	1.27	68.05	2.18
PL3	37°C-32°C-32°C	20.05	1.74	2.16	0.45	17.96	1.83	59.83	3.24

	sgRNA	CAMK-CR2							
	ssODN	C-CR2							
	ssODN Input	30 pmol							
Group	Temperature	(PctPerfectOligo)		(PctEditedOligo)		(PctPartialOligo)		(PctNonOligo)	
		Average	StdErr	Average	StdErr	Average	StdErr	Average	StdErr
PL1	37°C-37°C-37°C	4.2	1.36	1.01	0.41	4.34	0.9	90.46	2.63
PL2	37°C-32°C-37°C	10.12	1.83	1.4	0.43	9.25	1.08	79.23	3.31
PL3	37°C-32°C-32°C	14.77	2.23	1.79	0.4	12.32	0.54	71.12	2.85

Supplementary Table 3 continued. Effects of 'cold shock' and ssODN HDR donor design on HDR efficiency at the CAMK2D locus in mc-iPSC as determined by NGS

	sgRNA	CAMK-CR2							
	ssODN	C-CR2-Asym							
	ssODN Input	10 pmol							
Group	Temperature	(PctPerfectOligo)		(PctEditedOligo)		(PctPartialOligo)		(PctNonOligo)	
		Average	StdErr	Average	StdErr	Average	StdErr	Average	StdErr
PL1	37°C-37°C-37°C	1.24	0.29	0.2	0.05	5.16	1	93.4	1.32
PL2	37°C-32°C-37°C	3.21	0.54	0.46	0.1	9.28	1.15	87.06	1.76
PL3	37°C-32°C-32°C	5.72	0.54	0.52	0.08	14.93	0.49	78.83	0.65

	sgRNA	CAMK-CR2							
	ssODN	C-CR2-Asym							
	ssODN Input	30 pmol							
Group	Temperature	(PctPerfectOligo)		(PctEditedOligo)		(PctPartialOligo)		(PctNonOligo)	
		Average	StdErr	Average	StdErr	Average	StdErr	Average	StdErr
PL1	37°C-37°C-37°C	1.37	0.21	0.23	0.06	4.94	0.43	93.46	0.67
PL2	37°C-32°C-37°C	5.61	0.71	0.79	0.35	15.75	1.65	77.85	1.14
PL3	37°C-32°C-32°C	9.84	0.49	0.85	0.09	23.7	2.13	65.61	2.39

Supplementary Table 4. Effects of 'cold shock' and ssODN HDR donor design on HDR efficiency at the TGFBR1 locus in mc-iPSCs as determined by NGS

	sgRNA	TR-CR2							
	ssODN	None							
	ssODN input	None							
Group	Temperature	(PctPerfectOligo)		(PctEditedOligo)		(PctPartialOligo)		(PctNonOligo)	
		Average	StdErr	Average	StdErr	Average	StdErr	Average	StdErr
PL1	37°C-37°C-37°C	0	0	0.41	0.2	0.14	0.07	99.45	0.25
PL2	37°C-32°C-37°C	0	0	0.72	0.36	0.08	0.03	99.19	0.39
PL3	37°C-32°C-32°C	0.15	0.15	0.42	0.18	0.17	0.06	99.26	0.22

	sgRNA	TR-CR2							
	ssODN	T-CR2							
	ssODN input	10 pmol							
Group	Temperature	(PctPerfectOligo)		(PctEditedOligo)		(PctPartialOligo)		(PctNonOligo)	
		Average	StdErr	Average	StdErr	Average	StdErr	Average	StdErr
PL1	37°C-37°C-37°C	44.3	8.24	3.37	1.18	11.97	2.22	40.37	7.07
PL2	37°C-32°C-37°C	44.68	5.62	2.92	0.51	11.74	1.2	40.66	4.22
PL3	37°C-32°C-32°C	50.31	12.2	3.65	0.64	11.91	4.14	34.14	9.13

	sgRNA	TR-CR2							
	ssODN	T-CR2							
	ssODN input	30 pmol							
Group	Temperature	(PctPerfectOligo)		(PctEditedOligo)		(PctPartialOligo)		(PctNonOligo)	
		Average	StdErr	Average	StdErr	Average	StdErr	Average	StdErr
PL1	37°C-37°C-37°C	37.96	3.31	2.72	0.59	12.25	1.14	47.07	4.63
PL2	37°C-32°C-37°C	43.9	3.68	2.72	0.22	11.89	0.87	41.5	4.15
PL3	37°C-32°C-32°C	53.99	10.39	2.85	1.06	7.5	2.49	35.68	7.17

Supplementary Table 4 continued. Effects of 'cold shock' and ssODN HDR donor design on HDR efficiency at the TGFBR1 locus in mc-iPSCs as determined by NGS

	sgRNA	TR-CR2							
	ssODN	T-CR2-Asym							
	ssODN input	10 pmol							
Group	Temperature	(PctPerfectOligo)		(PctEditedOligo)		(PctPartialOligo)		(PctNonOligo)	
		Average	StdErr	Average	StdErr	Average	StdErr	Average	StdErr
**PL1**	**37°C-37°C-37°C**	16.83	3.42	2.05	0.48	23.38	1.53	57.74	3.89
**PL2**	**37°C-32°C-37°C**	21.74	1.19	2.1	0.7	32.93	4.24	43.23	4.65
**PL3**	**37°C-32°C-32°C**	24.8	2.5	2.74	0.37	28.96	2.53	43.5	2.98

	sgRNA	TR-CR2							
	ssODN	T-CR2-Asym							
	ssODN input	30 pmol							
Group	Temperature	(PctPerfectOligo)		(PctEditedOligo)		(PctPartialOligo)		(PctNonOligo)	
		Average	StdErr	Average	StdErr	Average	StdErr	Average	StdErr
**PL1**	**37°C-37°C-37°C**	19.71	0.88	2.04	0.34	28	3.04	50.26	2.19
**PL2**	**37°C-32°C-37°C**	27.2	1.67	2.47	0.97	26.02	1.88	44.32	2.71
**PL3**	**37°C-32°C-32°C**	28.23	1.9	2.53	0.71	26.02	1.62	43.23	2.89

Supplementary Table 4 continued. Effects of 'cold shock' and ssODN HDR donor design on HDR efficiency at the TGFBR1 locus in mc-iPSCs as determined by NGS

	sgRNA	TR-CR3							
	ssODN	None							
	ssODN input	None							
Group	Temperature	(PctPerfectOligo)		(PctEditedOligo)		(PctPartialOligo)		(PctNonOligo)	
		Average	StdErr	Average	StdErr	Average	StdErr	Average	StdErr
PL1	37°C-37°C-37°C	0	0	2.49	0.41	1.98	0.28	95.54	0.33
PL2	37°C-32°C-37°C	0	0	3.55	0.58	0.99	0.21	95.45	0.38
PL3	37°C-32°C-32°C	0	0	3.93	0.75	1.23	0.3	94.85	0.57

	sgRNA	TR-CR3							
	ssODN	T-CR3							
	ssODN input	10 pmol							
Group	Temperature	(PctPerfectOligo)		(PctEditedOligo)		(PctPartialOligo)		(PctNonOligo)	
		Average	StdErr	Average	StdErr	Average	StdErr	Average	StdErr
PL1	37°C-37°C-37°C	11.63	0.55	3.45	0.96	27.21	2.27	57.72	3.32
PL2	37°C-32°C-37°C	14.58	1.55	5.03	0.47	30.1	3.07	50.29	3.58
PL3	37°C-32°C-32°C	15.26	2.04	4.72	0.38	31.61	3.5	48.41	3.58

	sgRNA	TR-CR3							
	ssODN	T-CR3							
	ssODN input	30 pmol							
Group	Temperature	(PctPerfectOligo)		(PctEditedOligo)		(PctPartialOligo)		(PctNonOligo)	
		Average	StdErr	Average	StdErr	Average	StdErr	Average	StdErr
PL1	37°C-37°C-37°C	7.07	1.93	3.86	0.73	26.59	6.11	62.49	6.66
PL2	37°C-32°C-37°C	8.93	2.44	4.32	1.02	33.43	7.69	53.32	5.39
PL3	37°C-32°C-32°C	10.02	2.2	3.66	0.62	35.27	6.99	51.05	5.08

Supplementary Table 4 continued. Effects of 'cold shock' and ssODN HDR donor design on HDR efficiency at the TGFBR1 locus in mc-iPSCs as determined by NGS

	sgRNA	TR-CR3							
	ssODN	T-CR3-Asym							
	ssODN input	10 pmol							
Group	Temperature	(PctPerfectOligo)		(PctEditedOligo)		(PctPartialOligo)		(PctNonOligo)	
		Average	StdErr	Average	StdErr	Average	StdErr	Average	StdErr
**PL1**	**37°C-37°C-37°C**	6.42	1.52	2.79	0.67	24.83	2.61	65.96	2.71
**PL2**	**37°C-32°C-37°C**	8.67	1.58	3.65	0.74	27.55	2.12	60.14	2.32
**PL3**	**37°C-32°C-32°C**	8.46	1.25	3.34	0.27	28.35	2.11	59.85	1.75

	sgRNA	TR-CR3							
	ssODN	T-CR3-Asym							
	ssODN input	30 pmol							
Group	Temperature	(PctPerfectOligo)		(PctEditedOligo)		(PctPartialOligo)		(PctNonOligo)	
		Average	StdErr	Average	StdErr	Average	StdErr	Average	StdErr
**PL1**	**37°C-37°C-37°C**	4.9	1.54	4.15	1.14	33.81	4.98	57.14	4.63
**PL2**	**37°C-32°C-37°C**	6.49	1.88	3.32	0.74	30.4	2.34	59.78	2.78
**PL3**	**37°C-32°C-32°C**	5.5	0.34	3.88	1.17	33.42	6.47	57.21	6.15

Supplementary Table 5. HDR efficiencies at various temperatures at the CAMK2D locus in HEK293T cells as determined by ddPCR

Group		PL1		PL2		PL3	
Conditions		37°C-37°C-37°C		37°C-32°C-37°C		37°C-32°C-32°C	
sgRNA		CAMK-CR1	CAMK-CR2	CAMK-CR1	CAMK-CR2	CAMK-CR1	CAMK-CR2
ssODN input	None	0.01±0	0.02±0	0±0	0.01±0	0±0	0±0
	10 pmol	1.12±0.06[a]	1.51±0.07[c]	7.13±0.21[a]	10.45±0.21[c]	6.65±0.06 [a]	10.45±0.31[c]
	30 pmol	0.57±0.02[b]	0.81±0.04[d]	3.94±0.05[b]	6.09±0.19[d]	3.40±0.06[b]	7.10±0.4[d]

The experiments were carried out at different temperatures and HDR efficiencies were determined by ddPCR using probes specific for wild type and mutant sequences at the CAMK2D locus. Data presented as mean percentage of mutation containing droplets ± SEM from three biological replicates. The significance of HDR efficiency difference among the three temperature conditions for each sgRNA and ssODN treatment were analyzed by one way ANOVA.(a,b,c,d: one way ANOVA $P<0.0001$, follow-up Dunnett's multiple comparison, PL2 versus PL1: $P = 0.0001$, PL3 versus PL1: $P = 0.0001$)

Supplementary Table 6. 'Cold shock' enhances HDR efficiency at the CAMK2D locus in HEK293T cells as determined by NGS

	sgRNA	CAMK-CR1							
	ssODN	None							
	ssODN Input	None							
Group	Temperature	(PctPerfectOligo)		(PctEditedOligo)		(PctPartialOligo)		(PctNonOligo)	
		Average	StdErr	Average	StdErr	Average	StdErr	Average	StdErr
**PL1**	**37°C-37°C-37°C**	0	0	0.06	0.01	0.19	0.01	99.75	0.01
**PL2**	**37°C-32°C-37°C**	0	0	0.16	0.01	0.08	0.01	99.76	0.01
**PL3**	**37°C-32°C-32°C**	0	0	0.16	0	0.11	0.01	99.73	0.01

	sgRNA	CAMK-CR1							
	ssODN	C-CR2							
	ssODN Input	10 pmol							
Group	Temperature	(PctPerfectOligo)		(PctEditedOligo)		(PctPartialOligo)		(PctNonOligo)	
		Average	StdErr	Average	StdErr	Average	StdErr	Average	StdErr
**PL1**	**37°C-37°C-37°C**	0.17	0.06	0.10	0.03	0.6	0.03	99.12	0.1
**PL2**	**37°C-32°C-37°C**	1.93	0.36	0.91	0.09	3.55	0.5	93.6	0.94
**PL3**	**37°C-32°C-32°C**	1.61	0.28	0.72	0.06	3.07	0.53	94.59	0.87

	sgRNA	CAMK-CR1							
	ssODN	C-CR2							
	ssODN Input	30 pmol							
Group	Temperature	(PctPerfectOligo)		(PctEditedOligo)		(PctPartialOligo)		(PctNonOligo)	
		Average	StdErr	Average	StdErr	Average	StdErr	Average	StdErr
**PL1**	**37°C-37°C-37°C**	0.09	0.02	0.02	0	0.41	0.06	99.49	0.08
**PL2**	**37°C-32°C-37°C**	1.36	0.17	0.45	0.06	1.77	0.15	96.42	0.36
**PL3**	**37°C-32°C-32°C**	0.99	0.18	0.19	0.03	1.35	0	97.46	0.19

Supplementary Table 6 continued. 'Cold shock' enhances HDR efficiency at the CAMK2D locus in HEK293T cells as determined by NGS

	sgRNA	CAMK-CR2							
	ssODN	None							
	ssODN Input	None							
Group	Temperature	(PctPerfectOligo)		(PctEditedOligo)		(PctPartialOligo)		(PctNonOligo)	
		Average	StdErr	Average	StdErr	Average	StdErr	Average	StdErr
PL1	37°C-37°C-37°C	0	0	0.01	0	0.29	0.04	99.7	0.05
PL2	37°C-32°C-37°C	0	0	0.12	0.03	0.34	0.01	99.54	0.04
PL3	37°C-32°C-32°C	0	0	0.13	0.03	0.29	0.04	99.58	0.07

	sgRNA	CAMK-CR2							
	ssODN	C-CR2							
	ssODN Input	10 pmol							
Group	Temperature	(PctPerfectOligo)		(PctEditedOligo)		(PctPartialOligo)		(PctNonOligo)	
		Average	StdErr	Average	StdErr	Average	StdErr	Average	StdErr
PL1	37°C-37°C-37°C	0.13	0.03	0.07	0.02	0.74	0.1	99.06	0.15
PL2	37°C-32°C-37°C	2.39	0.49	0.71	0.29	8.12	2.66	88.78	3.43
PL3	37°C-32°C-32°C	1.78	0.37	0.55	0.08	5.84	1.55	91.83	1.98

	sgRNA	CAMK-CR2							
	ssODN	C-CR2							
	ssODN Input	30 pmol							
Group	Temperature	(PctPerfectOligo)		(PctEditedOligo)		(PctPartialOligo)		(PctNonOligo)	
		Average	StdErr	Average	StdErr	Average	StdErr	Average	StdErr
PL1	37°C-37°C-37°C	0.19	0.03	0.02	0.02	0.44	0.02	99.36	0.04
PL2	37°C-32°C-37°C	2.28	0.79	0.51	0.20	4.77	1.94	92.44	2.68
PL3	37°C-32°C-32°C	2.34	1.00	0.34	0.14	3.18	0.77	94.14	1.88

Supplementary Methods

Additional Transfection Methods.

For mc-iPSCs lipid based DNA transfections, cells were seeded in Matrigel coated 24-well plates at $1x10^5$ cells per well in mTeSR media one day prior to transfection. On the day of transfection the media was replaced with 0.5 ml fresh media. 1 µg of pX458-CRISPR DNA was diluted in 50 µl OptiMEM medium followed by the addition of 2 µl DNA-In® Stem or DNA-In® CRISPR transfection reagent (MTI-GlobalStem). For homology directed repair experiments, ssODNs were re-suspended in water at 10 µM and various amounts of ssODNs were added to the DNA/media mixture before lipid addition. The samples were gently mixed and incubated at room temperature for 15 min. The entire mixture was then added to the cells drop by drop and the plates were incubated at 37°C in a 5% CO_2 incubator and cells harvested for genomic DNA extraction 48h later.

For mc-iPSCs, IVT sgRNA and Cas9 nuclease lipid based transfection, the procedure was similar to the DNA transfection with minor modifications {Liang, 2015 #164}. Specifically, 480 ng of IVT sgRNA and 2 µg of Cas9 nuclease were first mixed in 50 µl OptiMEM medium and keep at room temperature for 10 min to form stable RNP complexes followed by the addition of 2.5 µl mRNA-In Stem® or Edit-Pro™ (MTI-GlobalStem). For homology directed repair experiments, ssODNs were re-suspended in water at 10 µM and various amount of ssODNs were added to the complex before lipid addition. 100 ng of GFP mRNA was also spiked into each mixture to monitor the transfection efficiency. Plates were incubated at 37°C in a 5% CO_2 incubator and the cells were harvested for genomic DNA extraction 48h later.

For mc-iPSCs DNA nucleofection with or without ssODN, mc-iPSCs were first cultured in Matrigel-coated 10 mm dishes until 60-70% confluent. The cells were washed with PBS and treated with 3 ml of Accutase (Thermo Fisher Scientific) at 37°C for 5-8 min until all cells were dissociated. The cells were re-suspended in mTeSR media and counted. The cells were then transferred to 15 ml tubes and spun down at 80g for 5 min. The supernatant was removed and the cells were re-suspended in P3 or P4 Nucleofection solution (Lonza, Basel Switzerland) at $1x10^7$ cells per ml. 20 µl of the cell suspension was transferred to Eppendorf tubes and 1 µg of pX458-CRISPRs were added to each tube. For homology directed repair experiments, ssODNs were re-suspended in water at 10 µM and various amounts of ssODNs were also added to the mixture. The suspension was then transferred to each well of 8 well strip (Lonza, Basel Switzerland) with care to avoid generating bubbles and electroporated using Amaxa™ 4D-Nucleofector™ (Lonza, Basel Switzerland) with program CM-113 or CE-118. The nucleofected cells were directly plated into Matrigel-coated 24-well plates which contained 500 µl pre-warmed mTeSR media with 10 µM ROCK Inhibitor Y-27632. Plates were incubated at 37°C in a 5% CO_2 incubator and the cells harvested for genomic DNA extraction 48h later.

For mc-iPSCs IVT sgRNA and Cas9 nuclease nucleofection, the procedure was similar to DNA nucleofection with minor modifications. Specifically, 480 ng of IVT sgRNA and 2 µg of Cas9 nuclease were first mixed together in OptiMEM medium to final volume of 5 µl and keep at room temperature for 10 min to form stable RNP complexes. For homology directed repair experiments, ssODNs were re-suspended in water at 10 µM and various amounts of ssODNs were also added to the complex. The complex was then transferred to 20 µl of cell suspension in P3 or P4 nucleofection solution and electroporated using Amaxa™ 4D-Nucleofector™ with program CM-113 or CE-118 as described above.

For HEK293T IVT sgRNA/ Cas9 mRNA lipid based transfection, the same procedure was followed as described for transfection of mc-iPSCs except that the HEK293T cells were seeded in 24 well tissue culture plate in Dulbecco's modified Eagle medium (DMEM) supplemented with 10% FBS and 50 units/ml penicillin-streptomycin (Thermo Fisher Scientific). For Genomic DNA extraction from HEK293T cells, similar procedures were followed as mc-iPSCs except that 250 µl of 0.25%Trypsin-EDTA (Thermo Fisher Scientific) was used to dissociate the cells from the wells.

Immunocytochemistry of 'cold shocked' mc-iPSCs. mc-IPSC's were plated on Matrigel coated 24 well plates at $5x10^4$ cells per well in mTeSR media and divided into four groups (PL1-PL3). Group PL1 was maintained at 37°C for 72h. Group PL2 was incubated at 37°C for 24h, then 'cold-shocked' at 32°C for 24h, and then returned to 37°C for 24h. Group PL3 was incubated at 37°C for 24h, and then 'cold-shocked' at 32°C for 48h.. The cells were then fixed and stained with pluripotency specific antibodies according to the conditions described in the Human ES/iPS Cell Characterization Kits (SABxxx-1, System Biosciences). Briefly, the media was aspirated and the plates were gently washed twice with 0.5 ml PBS per well. The cells were then fixed with 0.5 mL 4% paraformaldehyde in PBS for 20 min at room temperature and then washed three times with 0.5 ml PBS . The cells were blocked and permeabilized with 0.5 ml of 0.1% Triton X-100 in PBS plus 10% normal goat serum solution (Thermo Fisher Scientific, Cat# 50-062Z) for 1h at room temperature. The plates were washed once with PBS and stained with 100 µl of primary antibody at 1:100 dilution in antibody dilution buffer (0.1% Triton X-100 in PBS plus 3% normal goat serum solution) at 4°C overnight. Primary antibodies were purchased from System Biosciences: SSEA3 (Cat. No. SAB-102A-1), Nanog (Cat. No. SAB-103A-1) and Oct4 (Cat. No. SAB-105A-1). The next day, the primary antibodies were removed and the plates were washed alternatively with PBST (0.1% Triton X-100 in PBS) and PBS for 5 times each and then incubated with 180 µl of secondary antibody at 1:800 dilution in antibody dilution buffer at room temperature for 1h. The secondary

antibody used for SSEA3 was Alexa Fluor 488-conjugated goat anti-Rat IgM (A-21212, Thermo Fisher Scientific), and the secondary antibody used for Nanog and OCT4 was Alexa Fluor 488-conjugated goat anti-Rabbit IgG (A-11034,Thermo Fisher Scientific). The plates were then washed alternatively with PBST and PBS for 5X each and stained with 200 µl Hoechst (H3570, Thermo Fisher Scientific) at 5 µg/ml for 5 min. The plate were washed 2X with PBS and imaged using a Keyence BZ-X710 Florescence microscope.
